# Adultcentrism in the moral instruction of *Di Zi Gui*: proposed psychological mechanisms and implications for children’s autonomy

**DOI:** 10.3389/fpsyg.2026.1989993

**Published:** 2026-10-16

**Authors:** Hui Liu, Pinghua Liu

**Affiliations:** 1 School of Liberal Arts, Shenzhen City Polytechnic, Shenzhen, China; 2 General Education College, Guangdong University of Science and Technology, Dongguan, China

**Keywords:** adultcentrism, Bleak Pedagogy, children’s autonomy, *Di Zi Gui*, moral internalization, Philosophy for Children, psychological control

## Abstract

*Di Zi Gui*, a Qing-era moral primer, is discussed in contemporary Chinese family education, school moral education, and classical-recitation initiatives. Existing accounts document heterogeneous uses but do not establish their current prevalence or effects on children’s autonomy. This Hypothesis and Theory article develops a conditional model through close reading of 34 purposively selected precept groups from one modern working edition and comparison with psychological research from other contexts. Textual evidence, theoretical inference, and predictions requiring empirical testing are distinguished throughout. Adultcentrism identifies a possible asymmetry in interpretive authority; self-determination theory supplies the core distinction between controlled compliance and endorsed commitment. The proposed developmental focus is middle childhood, approximately 8–12 years, without assuming that findings from other age groups transfer directly. Three candidate pathways concern the permitted form of action, the motivational basis of conformity, and the legitimacy and expression of affect: behavioral standardization, compliance reinforcement, and emotional suppression through moral absolutization. Their operation is hypothesized to depend on enactment and children’s acceptance, negotiation, reinterpretation, selective appropriation, strategic compliance, or resistance. Autonomy is treated as a culturally situated, relational capacity, not as independence from family obligations. Five propositions specify an exposure-only baseline comparison, distinct mediators and moderators, and autonomy-related outcomes. Philosophy for Children is considered a possible moderator only when children’s contributions can consequentially alter interpretation or practice. The article offers an auditable interpretive framework, not evidence of established mechanisms: it contains no original participant data, direct observations of current teaching, or estimates of prevalence or effect size. Its contribution is a differentiated research agenda for testing how the same text may be enacted and understood differently.

## 1 Introduction

### 1.1 The contemporary revival of *Di Zi Gui*

Published accounts describe a revival of *Di Zi Gui* through recitation networks, family programs, schools, and traditional-culture initiatives, without establishing the prevalence of these uses today (Huang, 2011; Zhang, 2019). The debate concerns a Qing-era primer, not a pre-Qin Confucian classic: its received text organizes instruction around filial and fraternal conduct, bodily comportment, truthful speech, care, and learning (Li, 2005). Supporters value the translation of virtues into everyday conduct; critics question whether concrete rules can be enacted as rigid, adult-defined requirements. This tension invites a psychological question about how inherited prescriptions are interpreted and received, rather than a direct inference from historical wording to contemporary child outcomes.

This revival amplified both the text’s educational appeal and its contested status. Supporters emphasize its mnemonic form and its concrete guidance concerning filial care, honesty, courtesy, self-discipline, and everyday responsibility (Qin, 2018; Zhang, 2019). Critics focus on prescriptions that can be interpreted as subordinating children’s judgment, conduct, and emotional expression to senior authority, especially when recitation is compulsory or individual precepts are detached from historical context (Shen, 2018; Zhang, 2019). Yet the resulting debate is often framed too narrowly as a choice between cultural preservation and rejection. The more analytically useful questions concern how the text is selected, interpreted, taught, enforced, and received. A rule that supplies predictable behavioral structure in one setting can be enacted as non-negotiable psychological control in another. Conversely, rejecting compulsory study does not require rejecting moral education, intergenerational responsibility, or the possibility of critically reconstructing inherited ethical resources (Zhang, 2019).

### 1.2 Limitations of existing scholarship

Existing scholarship can be grouped into four overlapping bodies. Textual and historical studies examine authorship, editions, structure, sources, and circulation (Huang, 2011; Zhang, 2019). Research on family instruction and Confucian ethics emphasizes filiality, trustworthiness, benevolence, and the conversion of broad virtues into everyday practices (Qin, 2018). A third body debates contemporary suitability, including selective reinterpretation versus literal or compulsory application (Shen, 2018; Zhang, 2019). A smaller fourth body considers developmental implications or educational implementation. Shek and Law (2019) compare the primer with contemporary developmental knowledge but leave actual child outcomes to future research. One single-class action study reported participant and parent perceptions after a bundled responsibility program; its multicomponent design and lack of a comparison group prevent attribution to the text itself (Lin, 2018). The abstract of Yao and Wong (2021) reports associations among an intervention, prosocial behavior, peer relations, and teacher-student relations, but the design and outcome details require full-text verification.

Across these bodies of work, two contrary inferential shortcuts recur. Favorable accounts can treat visible compliance or prosocial conduct as evidence of successful moral internalization. Critical accounts can move directly from severe or hierarchical wording to claims of psychological injury. Both shortcuts collapse distinctions that are central to psychological explanation. Prosocial conformity and autonomous moral internalization are related but conceptually distinct outcomes. Self-determination theory, for example, differentiates externally regulated and introjected conduct from identified or more fully integrated regulation (Deci and Ryan, 2000). Likewise, textual affordances, adult interpretations, pedagogical practices, children’s meaning-making, and psychological outcomes occupy different evidentiary levels. What remains underdeveloped is a mechanism-focused account explaining how specific enactment conditions could connect moral micro-rules with different qualities of motivation, emotion, judgment, and participation–while preserving counterexamples, alternative interpretations, and the possibility of positive outcomes.

### 1.3 Research questions, thesis, and contributions

Adultcentrism provides a framework for this task when used precisely. It refers to adults’ tendency to interpret children’s capacities and concerns through a biased adult standpoint, not to adult responsibility or guidance as such (Petr, 1992; Biswas et al., 2024). It differs from adultism as institutionalized domination, ageism as age-based prejudice, and authoritarianism as a broader orientation toward hierarchy. Recent adultcentrism and Bleak Pedagogy research–formerly using Black Pedagogy in existing measure titles–has begun to operationalize adult-centered assumptions and harsh practices while examining how adult authority can render children’s perspectives epistemically secondary (Florio et al., 2020a,b, 2022; Luo and Yang, 2026; Santana, 2026).

Against this background, the article asks four questions: (RQ1) Which textual features of selected *Di Zi Gui* precepts can afford adultcentric interpretation or enactment under specified pedagogical conditions? (RQ2) Through which psychological pathways might such enactment shape the quality of children’s reasoning, motivation, emotion, and participation? (RQ3) How might the proposed mechanisms converge across epistemic, motivational-affective, and expressive-behavioral autonomy? (RQ4) How might Philosophy for Children reconstruct the teaching of *Di Zi Gui* while preserving ethically valuable resources?

The central thesis is conditional: rules and adult guidance do not inherently undermine autonomy. The primary empirical hypothesis is that enactment conditions and children’s reception will predict autonomy-related outcomes beyond exposure to *Di Zi Gui* alone. Adultcentrism specifies a possible asymmetry in interpretive authority, while self-determination theory distinguishes pressured compliance from endorsed commitment. Whereas Luo and Yang (2026) critique the developmental ontology that makes the child an incomplete adult and Santana (2026) proposes an adultcentric-bias hypothesis for teachers’ emotional violence, this article locates a testable intermediate account between a particular text, its enactment, children’s meaning-making, and autonomy. The three pathways developed below are theoretical inferences, not mechanisms established in *Di Zi Gui* learners. The analytical focus is middle childhood, approximately 8–12 years; extending the model to younger children or adolescents requires further developmental specification. Contemporary enactment, rather than Li Yuxiu’s intentions or Confucian civilization as a whole, is the object of the proposed research.

## 2 *Di Zi Gui* as text and pedagogical practice

### 2.1 Historical formation and textual status

The object examined here is the received *Di Zi Gui*, not “Confucian culture” in the aggregate. The working text is the version printed in the 2005 Zhonghua Book Company volume *Di Zi Gui* and Longwen Bianying, edited by the Beijing Sihai Classics Cultural Communication Center. It presents a compact Qing-period primer attributed to Li Yuxiu under five printed divisions: General Preface (总叙), Filiality at Home and Fraternal Respect Outside (入则孝，出则悌), Carefulness and Trustworthiness (谨而信), Universal Care and Association with the Humane (泛爱众而亲仁), and Learning When One Has Energy to Spare (行有余力，则以学文) (Li, 2005, pp. 3–11). This article does not reconstruct the full transmission genealogy because the available sources do not independently establish each reported change of title, attribution, and revision.

This distinction matters theoretically. Although the primer draws its organizational vocabulary from earlier Confucian formulations concerning conduct “at home” and “outside,” it is neither a pre-Qin source nor equivalent in textual status to the Analects or Mencius. Its present visibility also does not establish uninterrupted historical dominance. The evidence available for this study instead supports a limited conclusion: a mnemonic primer with a history of circulation in private educational settings acquired expanded authority through modern recitation networks and educational projects (Huang, 2011; Zhang, 2019). Consequently, claims about the primer cannot be generalized to all Confucian ethics, all Chinese education, or all intergenerational relations.

### 2.2 The moral architecture of the primer

The text’s pedagogical power lies in its conversion of broad relational virtues into observable micro-conduct. It links filiality to prompt responses, advance notice of departures, care of the body, and attentiveness to parents’ preferences; fraternal respect to precedence in eating, sitting, and walking; carefulness to dress, posture, and domestic routines; trustworthiness to restrained and accurate speech; and learning to practice, reading, note-taking, and inquiry. Three-character phrasing makes these prescriptions memorable, while repeated imperatives–respond, listen, do not act, speak softly–make performance publicly inspectable (Li, 2005; Qin, 2018).

That architecture is ethically double-sided. Abstract values become actionable rather than rhetorical, and a child is offered a repertoire of concrete practices through which care or reliability can be expressed. Yet the same compression minimizes deliberative information: who decides whether a command is justified, when obligations conflict, whether the norm is reciprocal, and what exceptions safety or fairness require. In “When parents call, respond without delay; when they give an instruction, do not be lax in carrying it out” (父母呼，应勿缓；父母命，行勿懒), the directive form and identity of the authority holder are explicit, while the reason for the instruction and the scope of legitimate refusal are not (Li, 2005, p. 3, author’s translation). This is a textual affordance for hierarchical enactment, not evidence that every reader accepts the line literally or that every instructor enforces it coercively.

The same concreteness also underlies a constructive reading. Huang argues that a contemporary interpretation should be “grounded in children’s everyday lives” (2011, p. 49, author’s translation). That criterion shifts attention from reciting a behavioral form to examining whether it actually serves care, safety, truthfulness, or coordination in a child’s present circumstances.

### 2.3 Ethical resources, contextual norms, and points of tension

The primer is not ethically uniform. Some passages provide strong resources for contemporary reconstruction. “All people should be treated with care” (凡是人，皆须爱) extends moral concern beyond kinship (Li, 2005, p. 8, author’s translation). “Before doing something to another, first ask yourself; if you would not want it, stop at once” (将加人，先问己；己不欲，即速已) articulates reciprocity (Li, 2005, p. 9, author’s translation). Most importantly for the present argument, “If power makes people submit, their hearts do not assent; if reason convinces them, only then is there no objection” (势服人，心不然；理服人，方无言) explicitly contrasts coercive power with reason-giving (Li, 2005, p. 9, author’s translation). The learning section likewise directs the learner who has doubts to record them and seek clarification: “When doubts arise, note them down; consult someone and seek the exact meaning” (心有疑，随札记；就人问，求确义; Li, 2005, p. 10, author’s translation). These lines complicate any claim that the text admits only silence or force.

Other prescriptions are better understood as historically situated conventions: precedence by age in eating or seating, lowered speech before elders, restrained talk, fixed routines, and rules of clothing or posture. Such norms may express courtesy or provide predictable structure, but their ethical standing depends on context, proportionality, reciprocity, and the child’s developing competence. Still other passages create sharper tensions with contemporary commitments to bodily safety and voice. “When parents give an instruction, do not be lax in carrying it out”; “when parents admonish, accept the admonition compliantly”; and the injunction to continue remonstrating even if struck, “harbor no resentment,” can be interpreted as making compliance and endurance constitutive of the child’s moral identity (Li, 2005, pp. 3–4, author’s translation). That interpretation is not inevitable: the remonstrance passage also recognizes that parents can be wrong and that children can address the wrong. The tension lies in coupling this limited critical role with an apparently unilateral duty to endure adult retaliation.

This mixture is why Qin’s recommendation that the text “should be approached dialectically” is analytically useful (2018, p. 36, author’s translation). Selective praise cannot dissolve the severe passages, while selective condemnation cannot erase the primer’s resources for reciprocity, reason, and inquiry.

### 2.4 From wording to outcome: six analytical levels

The central evidentiary safeguard is to distinguish six levels. The text supplies linguistic and normative affordances; adults select and interpret those affordances; pedagogical enactment determines whether they are accompanied by explanation, dialogue, rewards, shame, or punishment; children receive, negotiate, identify with, reinterpret, or resist what is taught; psychological mechanisms describe hypothesized processes within those encounters; and outcomes concern observable changes in judgment, motivation, emotion, relationships, or action. Neither adult intention nor textual form determines children’s meaning-making. An imperative at the first level cannot by itself establish a mechanism at the fifth or harm at the sixth.

The implementation evidence is limited. Lin’s (2018) Taiwanese action study describes an 11-week responsibility program in one third-grade class, combining *Di Zi Gui* materials with other teaching activities. Its single-class, multicomponent design cannot isolate a textual effect. Shek and Law (2019) offer a conceptual developmental appraisal, not an outcome study. The abstract of Yao and Wong (2021) reports an intervention involving four fifth-grade classes (*N* = 242), prosocial behavior, and peer and teacher–student relationships. Its allocation, fidelity, and causal strength cannot be assessed from the abstract, and those outcomes are not autonomy measures. These sources motivate investigation; they do not validate the pathways proposed here.

This distinction also clarifies the educational debate. Shen characterizes a recurrent problem in traditional moral instruction as proceeding “in a one-way, monologic manner” (2018, p. 10, author’s translation), while Zhang concludes that learning *Di Zi Gui* “should not be compulsory” (2019, p. 164, author’s translation). These are claims about pedagogy and curriculum authority, not proof that every use is coercive. They direct analysis toward the implementation conditions under which a text becomes compulsory, dialogical, selective, or revisable.

### 2.5 Documented contexts and limits of contemporary claims

Zhang’s (2019, pp. 146–148) documentary overview reports uses across schools, classical-recitation initiatives, community or training settings, and families. It describes a broad range of audiences, not a representative sample or a current census. Lin’s (2018) third-grade action study provides a more specific classroom example: recitation and explanation were combined with stories, role play, and responsibility-related activities. The available Yao and Wong (2021) abstract identifies fifth-grade classes but does not supply enough detail to characterize instructional format or voluntariness. These accounts establish that more than one setting and teaching format has been described; they do not establish which format is typical today.

Neither recitation nor institutional enrollment by itself indicates coercion. Relevant distinctions include whether participation is required, whether the child can withdraw or disagree without penalty, and whether recitation is accompanied by explanation, discussion, or conduct monitoring. Those details are not consistently available in the present sources. Family and school enactment should also be distinguished analytically: pressures involving attachment or household belonging may differ from grading, public performance, or peer visibility, although either setting can combine them. These contrasts, and the instructional scenarios in Sections “5 Three proposed adultcentric psychological mechanisms” and “7 Dialogical reconstruction through philosophy for children,” are constructed possibilities to be investigated, not observations of typical current practice.

### 2.6 Developmental scope

The initial scope is middle childhood, approximately 8–12 years, chosen to make the empirical agenda more specific and to connect cautiously with the primary-school contexts described above. It is not an age range in which the proposed mechanisms have been demonstrated. Exact age and language comprehension should be recorded; assent, interpretation, and motivation should be elicited through concrete situations and child-accessible tasks. Dependence on adult scaffolding is not itself an autonomy deficit. Earlier-childhood applications would require different elicitation and observation strategies, while adolescent extensions would need to consider changing relations with peers and authority. Findings from young-child, adolescent, or retrospective adult studies are therefore used as theoretical background, not interchangeable evidence for this target population.

## 3 Theoretical framework

### 3.1 Adultcentrism and Bleak Pedagogy

Adultcentrism names a patterned bias in which adult experience becomes the default measure of children’s capacities, interests, and credibility. Petr (1992) introduced the construct to expose practice that interprets children through adult assumptions rather than from their own standpoint. More recent scale development treats it as empirically examinable rather than merely rhetorical (Florio et al., 2020a; Biswas et al., 2024). Its epistemic component grants adults presumptive authority over what children know and what counts as a good reason; its normative component treats the child principally as an incomplete future adult whose present perspective can be overridden. A child’s disagreement can then be read as immaturity before its substance is considered.

The concept must not be inflated. Adults often have more experience, bear legal and moral duties of protection, and sometimes must make urgent safety decisions. These asymmetries become adultcentric when they are converted into an exemption from explanation, a presumption that age guarantees correctness, or a standing entitlement to define the child’s interests without meaningful participation. Adultcentrism is therefore distinct from adultism, which more specifically names age-based structural domination; from ageism, a broader category of age prejudice; and from authoritarianism, a general orientation toward obedience and hierarchy. In this article it designates the cognitive-normative premise that can authorize particular educational practices.

Bleak Pedagogy identifies a more disciplinary register. Historical publications and the validated instrument retain the term “Black Pedagogy,” while the current research program uses “Bleak Pedagogy” to avoid the racialized implications of the older English label (Florio et al., 2020b; Luo and Yang, 2026; Santana, 2026). The construct covers practices through which adults seek to break, shame, frighten, or manipulate a child’s will while presenting control as necessary for the child’s good. Adultcentrism and Bleak Pedagogy are related but nonidentical: the former can legitimate the adult’s interpretive monopoly, whereas the latter describes harsh techniques that may follow from it. Not every adultcentric assumption becomes punitive practice, and not every rule is bleak. The critical transition occurs when a presumed developmental deficit authorizes disproportionate control.

### 3.2 Culturally situated and relational autonomy

Autonomy is not a culturally neutral label that can simply be applied to a historical moral tradition. Here it is an explicit contemporary analytical commitment: children should increasingly be able to understand reasons, articulate concerns, and endorse obligations within relationships. The historical vocabulary of filial duty does not necessarily divide personhood into the same categories. Neither a single Western conception of the independent individual nor a homogeneous Chinese relational self is assumed. Interpretations of obligation must be examined in their educational and historical circumstances, including the terms children and adults themselves use for care, respect, filiality, and dissent.

Individual independence concerns acting without reliance on others; autonomy in the self-determination account concerns volitional endorsement. They need not coincide. Chirkov et al. (2003) distinguish autonomy from individualism and independence, while Kağıtçıbaşı’s (2005) conceptual account separates agency from interpersonal distance. These distinctions make autonomy-with-relatedness intelligible, but they do not establish that the present criteria are universal or already valid for Chinese children. A child’s willing filial commitment can be autonomous even when it involves demanding obligations and interdependence. Conversely, choosing an apparently individual course under shame or threatened rejection need not be autonomous. Relational autonomy here concerns commitments sustained and revisable through care, reasons, and participation, rather than freedom from attachment.

Three outcome domains operationalize this commitment provisionally. Epistemic autonomy concerns questioning, judging reasons, and recognizing exceptions; motivational-affective autonomy concerns the quality of regulation and the ability to acknowledge one’s needs and feelings; expressive-behavioral autonomy concerns dissent, help-seeking, age-appropriate initiative, and participation. These are analytical domains, not a validated scale or developmental stage sequence. They must not be equated with independence, talkativeness, obedience, or prosocial behavior. For children aged approximately 8–12, assessments should permit concrete examples and supported explanation rather than require adult-like abstract reflection. Integrated regulation is not assumed to be a reliably distinguishable outcome at every age; P1 prioritizes external, introjected, and identified regulation.

The primer’s own resources also resist an autonomy–tradition binary: remonstrance, reciprocity, inquiry, and the contrast between power and reason can support criticism from within filial relationships (Section “2.3 Ethical resources, contextual norms, and points of tension”). Future interviews should explore whether children understand a practice as care, legitimate duty, pressure, or something else before imposing those categories. Participation rights provide an openly normative commitment, not additional psychological proof that a particular verse damages autonomy (United Nations, 1989; Committee on the Rights of the Child, 2009). Thus, cultural interpretation and the evaluation of coercion require argument and children’s perspectives, not an automatic inference from either family obligation or cultural difference.

### 3.3 Motivation, psychological control, and emotion socialization

Self-determination theory distinguishes not only how much motivation a person has but how conduct is regulated. External regulation depends on rewards, punishment, surveillance, or command. Introjected regulation brings pressure inside: action is driven by guilt, shame, contingent self-worth, or the demand to prove oneself good. Identified regulation occurs when a person recognizes a behavior’s value; more integrated regulation aligns that value with a coherent sense of self (Deci and Ryan, 2000). This continuum prevents visible obedience from serving as a proxy for autonomous internalization.

Psychological control is especially relevant because it targets the child’s inner world rather than merely specifying conduct. It intrudes on thoughts and feelings through guilt induction, shame, withdrawal of affection, and pressure on self-worth. Conditional regard is one form: approval or closeness is experienced as contingent on meeting the adult’s expectations. In retrospective correlational studies, parental conditional regard was associated with compliance but also with internal compulsion and emotional costs (Assor et al., 2004). Multi-informant adolescent research likewise distinguished parental conditional regard from autonomy support and linked it with resentment or compulsive engagement (Roth et al., 2009). These studies do not concern *Di Zi Gui* and do not establish what any particular verse causes. They justify a mechanism hypothesis: an instructor can attach filial language to conditional worth, thereby shifting a rule from behavioral structure toward psychological control.

Measurement work in mainland China also cautions against treating psychological control as culturally uniform. Zhu et al. (2023) developed a Chinese parental psychological-control measure through focus groups with 19 adolescents and validation with 963 seventh- and eighth-grade students. Their three-factor solution distinguished relational induction, harsh psychological control, and social-comparison shame; the factors did not show identical relations with adjustment indicators. The cross-sectional convenience sample cannot establish causal pathways or validate the present model, but it supports differentiating culturally articulated relational appeals from harsher intrusion and shame rather than labeling every invocation of family obligation as the same form of control.

The distinction is crucial. Behavioral structure communicates clear expectations, rationales, predictable consequences, and support for meeting them. It can protect safety and coordinate shared life. Psychological control pressures the child to think, feel, or value the self as the adult prescribes. Thus, a family rule requiring notification before leaving may be autonomy-supportive if it is justified by safety, applies reciprocally where appropriate, and expands as competence develops. The same rule becomes controlling when an unexplained delay is treated as proof that the child is unfilial or unworthy.

Emotion socialization provides a parallel distinction. Adults help children notice, name, interpret, express, and regulate emotion through modeling, discussion, and reactions to emotional displays (Morris et al., 2007). Regulation is not suppression. Expressive suppression is an effort to inhibit outward signs of an already activated emotional response (Gross, 1998); invalidation occurs when an emotional experience is dismissed, morally disqualified, or made unsafe to express. In a cross-sectional study of parents and young children, parental conditional regard regarding sadness suppression was associated with poorer sadness awareness and empathy, but its design does not warrant causal claims (Roth and Assor, 2010). The relevance here is conditional: when anger at mistreatment, fear of punishment, or disagreement is reclassified as filial failure, a lesson in composure can become pressure to conceal the information carried by emotion.

The explanatory framework is deliberately narrow. Adultcentrism identifies whose interpretation receives presumptive authority; self-determination theory specifies the central contrast between controlled and endorsed regulation. Psychological control and emotion invalidation supply candidate processes within this account, rather than additional overarching theories. Bleak Pedagogy names a relevant family of educational practices; relational autonomy defines the outcome orientation; participation rights state a normative constraint. P4C is a proposed pedagogical moderator, examined in Section “7 Dialogical reconstruction through philosophy for children,” not a further explanation of how the mechanisms originate. No self-efficacy pathway or developmental-stage theory is needed for the present model.

### 3.4 Children’s reception and meaning-making

Reception denotes how a child understands and responds to a particular enactment, not an automatic transmission of an adult’s intention. An initial typology distinguishes acceptance of the stated interpretation; negotiation of its application; reinterpretation of its meaning; selective appropriation of a value without its prescribed form; strategic public compliance without assent; and resistance through refusal, challenge, or seeking support. Invoking one precept against another is a potentially important form of reinterpretation: a child might question an order by appealing to the text’s contrast between power and reason. These are proposed analytical possibilities, not categories observed in this article, and they can coexist or change across episodes.

Reception should be elicited through private, age-appropriate explanations, alternative-case tasks, and observation of what happens after a child’s response. A quiet child cannot be classified as accepting, nor a compliant child as internally endorsing a rule, from conduct alone. Codes should describe interpretation and response to a specific episode, whereas later motivation and autonomy measures should assess distinct outcomes. This separation limits circularity in P1. Adult uptake also matters: an attempted negotiation that is ignored differs from one that changes an exception. Reception may therefore reshape enactment rather than merely occupy a one-way intermediate position in a diagram.

## 4 Analytical strategy

### 4.1 Corpus and units of analysis

This theory-guided conceptual analysis combines close reading with conditional mechanism mapping. The working edition is specified in Section “2.1 Historical formation and textual status.” The retained project ledger contains 34 purposively selected meaning-complete precept groups, identified as T01–T34; Table 1 illustrates 10 of them. Thirty-four is the size of this analytical selection, not a count of all possible units in the edition. The available audit trail does not establish exhaustive whole-edition segmentation or demonstrate that every unit was coded before examples were chosen. Accordingly, this article makes no claim to a systematic census, code frequencies, or representative coverage of the text.

**TABLE 1:** Theory-guided coding of selected *Di Zi Gui* precepts.

Precept and source	Moral value	Directive form	Authority relation	Sanction / emotional rule	Proposed conditional mapping	Potential autonomy implication	Proposed dialogical reconstruction
T01: 父母呼，应勿缓；父母命，行勿懒 (Li, 2005, p. 3)	Attentiveness; responsiveness	Commands	Parent initiates; child responds	No explicit sanction; promptness prescribed	Behavioral standardization; compliance reinforcement	Epistemic; expressive-behavioral	Ask when immediate response serves care and when a reasoned delay is legitimate; make responsiveness reciprocal.
T02: 父母教，须敬听；父母责，须顺承 (Li, 2005, p. 3)	Receptivity to guidance	Commands	Parent teaches/corrects; child listens/accepts	No explicit sanction; respectful, compliant reception	Compliance reinforcement	Epistemic; motivational-affective	Separate listening from agreement; require evidence and reasons from both parties.
T08: 身有伤，贻亲忧；德有伤，贻亲羞 (Li, 2005, p. 4)	Care; responsibility	Relational consequence	Child’s conduct evaluated through parents’ affect/status	Parental worry and shame	Compliance reinforcement	Motivational-affective	Discuss self-care and relational consequences without making the child responsible for adult worth or family honor.
T11: 谏不入......挞无怨 (Li, 2005, p. 4)	Persistence; filial commitment	Counsel plus prohibition	Child may remonstrate; retaliation remains textually unqualified	Being struck; resentment prohibited	Compliance reinforcement; emotional suppression	Motivational-affective; expressive-behavioral	Affirm remonstrance; reject violence; establish refusal, exit, disclosure, and help-seeking rights.
T14: 或饮食，或坐走，长者先，幼者后 (Li, 2005, p. 4)	Courtesy; order	Behavioral rule	Priority follows age	No explicit sanction	Behavioral standardization	Expressive-behavioral if generalized	Compare courtesy, need, disability, urgency, and reciprocal hosting across cases.
T18: 尊长前，声要低；低不闻，却非宜 (Li, 2005, p. 5)	Respect; audible speech	Command with qualification	Younger speaker adjusts before elder	Low voice qualified by audibility; no explicit penalty	Action-form standardization if rigidly enacted; affect pathway requires separate invalidation evidence	Expressive-behavioral	Distinguish courteous volume from silencing; identify when speaking clearly or urgently is responsible.
T25: 话说多，不如少；惟其是，勿佞巧 (Li, 2005, p. 7)	Truthfulness; restraint	Comparison and prohibitions	Not explicitly age-asymmetric	Excess and artifice discouraged	Behavioral standardization under literal quietness norm	Expressive-behavioral	Contrast gossip and interruption with correction, advocacy, disclosure, and necessary dissent.
T31: 将加人，先问己；己不欲，即速已 (Li, 2005, p. 9)	Reciprocity	Reason-like test	Potentially mutual	Self-restraint based on perspective-taking	Counter-resource	Possible support for autonomy; no observed outcome	Apply the test to adults and children; examine conflicts between preference, harm, and justice.
T32: 势服人，心不然；理服人，方无言 (Li, 2005, p. 9)	Reason over force	Contrast / reasons	Rejects power as sufficient	Marks outward submission as inadequate	Counter-resource to all three mechanisms	Epistemic; motivational-affective	Require reasons to be intelligible, contestable, and as mutually applicable as context permits.
T34: 心有疑，随札记；就人问，求确义 (Li, 2005, p. 10)	Inquiry; accuracy	Invitation to record and ask	Knowledgeable others assist inquiry	No explicit sanction	Counter-resource to interpretive monopoly	Epistemic	Permit child-generated questions and let inquiry revise, not merely retrieve, interpretation.

T identifiers refer to the 34-entry audit trail in Supplementary Material 1. This table illustrates 10 selected groups, not an exhaustive coding or a frequency distribution. The wording, an interpretive description, and a proposed conditional mapping are different evidence levels. Mechanism and autonomy columns require the enactment and reception criteria in Section “5 Three proposed adultcentric psychological mechanisms”; they do not report outcomes. English translations are the authors’ own; printed-page and scan-image locators, source checks, and edition-specific readings appear in Supplementary Material 1.

A unit is a meaning-complete precept group: paired instructions are kept together where their contrast or qualification is needed for interpretation. The retained selection includes directives about conduct, authority, correction, and emotion, together with passages that complicate an adultcentric reading through care, reciprocity, remonstrance, reasons, or inquiry. These criteria describe the present analytical set rather than a retrospectively invented prespecified search protocol. Supplementary Material 1 supplies all 34 entries, source locators, translations, conditional mappings, counter-readings, and source-check notes. It is a revision-stage audit trail, not participant data or a record of independent coding sessions.

For this revision, the wording and printed-page locators of all 34 selected passages were checked against clear images of the 2005 edition. The complete primer occupies printed pp. 3–11 (PDF images 13–21); the selected passages occur on pp. 3–10. Supplementary Material 1 records the edition’s readings and resolves the four previously qualified entries, T03, T05, T17, and T22. The T identifiers denote analytical groups, not the book’s circled recitation numbers. This check establishes transcription and location within one edition; it does not establish exhaustive coding, independent coder agreement, or psychological validity. See also Supplementary Material 2–6.

### 4.2 Coding and mechanism mapping

Nine dimensions guide the reconstruction of each entry: moral value; directive form; authority holder; reciprocity; sanction; emotional rule; provision of reasons; contextual qualification; and candidate mechanism. The first eight are descriptive or interpretive features of wording and context; the ninth is a conditional theoretical mapping. Inclusion in behavioral standardization requires an enactment that privileges one prescribed action form despite a reasoned alternative; compliance reinforcement requires evidence of rewards, sanction, conditional regard, or self-worth pressure sustaining conformity; emotional suppression through moral absolutization requires an affect to be morally disqualified and concealment or inhibition to follow. A command or silence alone satisfies none of these mechanism criteria.

Multiple mappings are permitted only when their separate conditions are specified. For example, a low-voice rule could constrain action form without threatening belonging or disqualifying anger; those latter mappings require additional evidence. Ambiguous wording is retained with a qualified reading rather than forced into one category. Remonstrance (T10–T11), care and reciprocity (T30–T31), reason over power (T32), and inquiry (T34) serve as counter-cases. The ledger does not document independent double coding, author-specific coding assignments, or a formal disagreement-resolution procedure; none is claimed, and no inter-rater reliability is reported. The supplied materials permit readers to inspect and contest the interpretive choices, but cannot establish the reproducibility of a procedure that was not recorded.

### 4.3 Interpretive safeguards

Three evidence levels delimit the argument. Textual evidence establishes wording and identifies an interpretive possibility; a conditional inference connects a specified enactment and child reception to a psychological construct; an empirical prediction states what future observations could support or challenge. Psychological findings from other tasks, cultures, and ages justify considering a pathway, not treating it as established in *Di Zi Gui* instruction. Section “5 Three proposed adultcentric psychological mechanisms” keeps these levels separate, and Section “8.2 Five propositions” specifies tests and disconfirming observations. The framework also retains competing accounts: rules can support safety and coordination, children can endorse filial commitments, and adults’ conduct or broader institutional conditions may outweigh the text. No mapping in the ledger is an estimate of a child outcome.

Generative AI assisted evidence organization, English-language drafting, editorial integration, and document preparation, including the September 2026 revision. It was not used as an independent coder or a source of participant observations. The authors remain responsible for checking sources, translations, interpretive decisions, and the final submitted text; the disclosure in the Statements section records the tool and its role.

## 5 Three proposed adultcentric psychological mechanisms

### 5.1 Behavioral standardization

Behavioral standardization concerns the prescribed form of action and the decision space left around it: a relational value is translated into a narrow, externally observable performance whose appropriateness is presumed in advance. *Di Zi Gui* repeatedly makes this translation available. Filial attentiveness becomes speed of response–“When parents call, respond without delay” (父母呼，应勿缓)–and parental direction becomes prompt execution–“when they give an instruction, do not be lax in carrying it out” (父母命，行勿懒; Li, 2005, p. 3, author’s translation). Respect becomes bodily precedence: “Whether eating, drinking, sitting, or walking, elders go first and juniors follow” (或饮食，或坐走，长者先，幼者后; p. 4, author’s translation). It also becomes vocal modulation: “Before elders, one’s voice should be low; but so low that it cannot be heard is also inappropriate” (尊长前，声要低；低不闻，却非宜; p. 5, author’s translation). The last qualification shows that the text is not indifferent to practical intelligibility, yet the rule remains organized around the younger person’s conduct before a senior.

Such specificity has genuine pedagogical advantages. It reduces abstraction, helps novices anticipate shared routines, and can make courtesy and care easier to practice. A prompt response may communicate attentiveness; giving notice before leaving can support family safety; turn-taking can coordinate collective life. A structured environment is not equivalent to psychological control. The critical question is whether the micro-rule is presented as a revisable expression of a value or as the value’s exhaustive and identity-defining form.

The conditional inference concerns an enactment in which an adult treats the script and its exceptions as beyond question. A child attending to another urgent task, managing sensory overload, or offering a reasoned delay might then be judged solely against the visible template. This is a constructed scenario, not a classroom observation. Its psychological significance would depend on reception: the child might accept the judgment, privately reject it, negotiate an exception, or reinterpret promptness as reciprocal attentiveness. Only evidence about those responses and subsequent opportunities for judgment could connect the scenario to an autonomy outcome.

The discriminating target of behavioral standardization is the permitted form of action. A candidate observation would be the rejection of a child’s equally safe, reasoned alternative solely because it departs from the authorized script, even when no emotional display or rewards is at issue. Repeatedly selecting the approved form without being able to explain its purpose could support the proposed pathway when independent reception evidence rules out strategic compliance. Conversely, flexible acceptance of reasoned alternatives would weaken this interpretation. Exact performance, courtesy, or memorization alone does not establish either the mechanism or reduced autonomy. This is a prediction about enactment and reception, not a psychological property demonstrated by T01 or T14.

The strongest counterargument is that novices need routines before they can deliberate well. That is plausible, especially for safety and coordination. The distinction is not between routines and autonomy but between scaffolding and permanent substitution. Structure is autonomy-supportive when adults explain its protective value, model the same value, allow age-appropriate choice, identify exceptions, and transfer decision authority as competence grows. It becomes standardizing when the observable form is moralized, exceptions remain adult privileges, and compliance is treated as sufficient evidence of virtue. The proposed mechanism therefore depends on enactment conditions: decontextualization, surveillance, adult control of exceptions, and evaluation of the child’s moral identity through micro-performance.

### 5.2 Compliance reinforcement

Compliance reinforcement differs in explanatory target: it concerns why conformity is secured and maintained through rewards, sanctions, shame, guilt, conditional belonging, or their anticipation. Some *Di Zi Gui* precepts make the desired response explicit: “When parents instruct, listen respectfully; when parents admonish, accept the admonition compliantly” (父母教，须敬听；父母责，须顺承; Li, 2005, p. 3, author’s translation). Here “compliantly” preserves the receptive and acquiescent force of 顺承 without adding a claim of absolute obedience. Elsewhere, bodily and moral condition are tied to relational consequences: “If the body is injured, one brings worry to one’s parents; if one’s moral conduct is harmed, one brings shame to one’s parents” (身有伤，贻亲忧；德有伤，贻亲羞; p. 4, author’s translation). These formulations can encourage consideration of how one’s conduct affects close others. They can also be enacted by making the child responsible for adult distress, family honor, or the continuation of affection.

Self-determination theory predicts different qualities of compliance depending on the perceived source of action (Deci and Ryan, 2000). An externally regulated child obeys to avoid punishment, reprimand, or loss of privilege. An introjected form of regulation operates when surveillance has moved inward: the child obeys to avoid feeling ashamed, guilty, or unworthy of being a good son, daughter, or student. Identified regulation would instead involve understanding why responsiveness, honesty, or care matters and choosing to enact that value. The visible behavior may be identical across these forms; the motivational organization is not.

An adultcentric pedagogy can reinforce external or introjected regulation in at least three ways. First, it equates adult satisfaction with moral correctness, so disagreement threatens evaluation or belonging. Second, it globalizes a disputed act into a judgment about the child’s relational identity–lazy, disrespectful, or unfilial. Third, it makes submission itself the test of virtue, irrespective of the command’s content. Conditional regard research supplies an analogous, not text-specific, evidentiary base. Adults’ approval made contingent on expected behavior has been associated with compliance alongside internal compulsion and emotional costs (Assor et al., 2004), while adolescent multi-informant research distinguishes such pressure from autonomy support (Roth et al., 2009). The studies are correlational and concern parenting domains other than this primer. They support the plausibility of a motivational pathway, not an estimate of the primer’s effect.

The passage on parental wrongdoing reveals the mechanism’s complexity. The child is instructed to remonstrate gently when parents err and, if the advice is not accepted, to try again when the parents are receptive (Li, 2005, p. 4). This grants the child some moral discernment and rejects the proposition that parental conduct is infallible. Yet the sequence concludes: “Even if struck, harbor no resentment” (挞无怨; p. 4, author’s translation). Under a historical or literary interpretation, “without resentment” may praise persistence, relational commitment, or self-command rather than authorize assault. Under an absolutized instructional interpretation, however, it can remove resentment and self-protection from the range of legitimate responses to adult violence.

This is where psychological control differs most clearly from reasonable discipline. A safety boundary specifies prohibited behavior and provides a proportionate consequence; it need not demand that the child approve of the adult or feel no anger. Psychological control reaches into allegiance and self-evaluation. If an adult uses “without resentment” to invalidate protest, threatens relational withdrawal, or requires gratitude after punishment, the child is pressed not only to comply but to disown the grounds of dissent. This would support a compliance-reinforcement interpretation only if the child experiences obedience as safer–emotionally and relationally–than independent appraisal.

The discriminating target of compliance reinforcement is the motivational basis of conformity, not its visible form. Candidate evidence would combine observed contingencies with a child’s account of acting to avoid disapproval, guilt, or loss of worth, assessed separately from later regulation outcomes. If a child gives the same response because the obligation is understood and endorsed, the observed compliance does not support this mechanism. The strongest alternative is active filial identification, possibly strengthened by consistent, caring guidance. Distinguishing the two requires private accounts and variation in contingencies; neither strictness nor parental expectations alone establishes psychological control.

### 5.3 Emotional suppression through moral absolutization

The third proposed mechanism concerns which emotions may be acknowledged or expressed when conformity is contested. Under this hypothesis, it could arise when a relational norm is treated as absolute and emotions that question it are morally disqualified. *Di Zi Gui* regulates not only action but the presentation of affect. A child whose parents do not show affection is instructed to remain exemplary in filiality (Li, 2005, p. 4). In mourning, grief is prescribed as sustained weeping and behavioral withdrawal (p. 4). Before elders, voice is lowered, while excessive talk is discouraged: “It is better to speak little than too much; say only what is true or fitting, and do not use glib or artful words” (话说多，不如少；惟其是，勿佞巧; p. 7, author’s translation). These passages can support loyalty amid relational difficulty, culturally meaningful mourning, composure, and truthful restraint. None inherently requires suppression.

Moral absolutization changes their operation by treating one emotional script as universally virtuous and alternative feelings as evidence of moral failure. Anger at unfair treatment can be renamed disrespect; fear can be treated as weakness or disobedience; ambivalence toward a harmful caregiver can be judged unfilial; and speech necessary for help-seeking can be dismissed as excessive. The issue is not that adults teach regulation. Emotion socialization necessarily includes guidance about timing, intensity, and expression (Morris et al., 2007). The issue is whether children can acknowledge an emotion, examine what it indicates, and select a safe expression, or must conceal it to protect an adult-defined moral identity.

Expressive suppression describes inhibiting the outward display of an activated emotional response (Gross, 1998). Emotion invalidation is broader: the experience is minimized, denied, or condemned. In the proposed pathway, invalidation supplies the moral reason for suppression. If a child learns that “good” children do not feel anger toward parents, the social cost of honest disclosure rises. Conditional regard can intensify the process because acceptance appears tied not only to conduct but to the “right” feeling. Roth and Assor’s (2010) cross-sectional study found associations between parental conditional regard concerning sadness suppression and young children’s sadness awareness and empathy. It neither tests the present text nor establishes direction of causation, but it underscores why the regulation-suppression distinction is psychologically consequential.

The phrase “Even if struck, harbor no resentment” is the clearest risk case because it joins harmful treatment to a prohibition on a negative relational response. Read literally and enforced by an adult, it can interfere with the recognition that anger and fear sometimes signal boundary violation. It may also compromise disclosure: a child who believes resentment itself is wrong has fewer safe words for naming harm. The inference remains conditional. The verse does not tell us how contemporary children understand the archaic line, whether instructors reject its literal use, or whether children resist it. It identifies an unusually strong affordance for an enactment that invalidates protective emotion.

The text also contains internal checks on absolutization. It distinguishes accidental from intentional wrongdoing–“Unintentional wrongdoing is called a mistake; intentional wrongdoing is called an evil” (无心非，名为错；有心非，名为恶; Li, 2005, p. 8, author’s translation)–and praises willingness to hear criticism (p. 8). Its contrast between coercion and reasons affirms that outward submission can coexist with inward nonassent (p. 9). These lines support attention to intention, correction, and the limits of force. They do not erase the more severe passages, but they provide resources for interpreting emotional protest as morally informative rather than inherently disloyal.

The discriminating target here is the legitimacy and expression of affect. A candidate observation would pair an adult’s moral disqualification of anger, fear, or sadness with the child’s reported concealment despite continuing to feel it. Mere quietness, low voice, or successful calming would not suffice. A child may follow a behavioral rule while safely expressing anger, or comply from guilt without hiding emotion; such cases separate this pathway from the other two. Acceptance of emotion with proportionate limits on action would weaken the moral-absolutization account. The text supplies possible interpretive material, while the sequence from invalidation to concealment remains an empirical hypothesis.

The three pathways therefore concern action form, reasons for conformity, and affective legitimacy, respectively. Their co-occurrence is possible, but a single indicator must not count as confirmation of all three. Future measurement should compare distinct-factor and collapsed models, include cases where only one criterion is met, and test whether each process adds explanatory information. Failure to distinguish the processes would count against the proposed three-mechanism structure, not merely justify relabeling the same behavior.

## 6 Implications for children’s autonomy

The proposed pathways need not correspond one-to-one with the three autonomy domains. A restriction on action form might affect interpretation or initiative; motivational pressure might affect commitment or voice; concealed emotion might limit the information available for judgment. These are cross-pathway hypotheses, not findings. The following sections identify possible outcome domains while leaving their associations with each process open to disconfirmation.

### 6.1 Epistemic autonomy

Epistemic autonomy is most directly at risk when adult status predetermines what counts as knowledge, a relevant question, or a permissible exception. Behavioral standardization supplies ready-made answers, while compliance reinforcement raises the cost of asking whether the rule fits. Repeated instruction to receive parental direction respectfully can be educationally defensible if listening initiates inquiry. It becomes epistemically restrictive when listening is defined as assent and the child’s counterevidence is treated as immaturity.

The risk is not simply reduced talk. It is a diminished opportunity to practice moral interpretation: distinguishing safety from convenience, care from deference, accident from intention, or justified authority from force. Over time, an adult monopoly over these judgments may make children dependent on external confirmation even when the text itself recognizes questioning. The directive to note doubts and seek exact meaning (Li, 2005, p. 10) provides a contrasting possibility. If “a knowledgeable person” is fallible and the inquiry can change the interpretation, the passage supports epistemic agency. If the learner may ask only to retrieve a predetermined answer, the form of inquiry remains adultcentric.

### 6.2 Motivational-affective autonomy

Motivational-affective autonomy concerns whether children can act from values they understand and endorse while remaining able to recognize their own emotional experience. Compliance reinforcement can shift behavior toward external or introjected regulation when punishment, adult disappointment, shame, or conditional belonging supplies the operative reason (Deci and Ryan, 2000; Assor et al., 2004). Emotional absolutization can deepen the constraint by classifying doubt or anger as proof of a defective relationship to the adult.

The alternative is not unregulated preference. A child can identify with filial care, truthful speech, or responsibility and still accept demanding obligations. The distinguishing questions are whether the value has been explained, whether the child can voice conflict, whether adult affection survives disagreement, and whether the rule can be revised in light of harm. Prosocial behavior therefore cannot serve as a stand-alone measure of this dimension. A child may help others from identification, rewards-seeking, guilt, fear, or a mixture of motives. Future research must assess regulation quality and emotional experience alongside conduct.

### 6.3 Expressive-behavioral autonomy

Expressive-behavioral autonomy is constrained when deference scripts generalize into habitual silence, avoidance of experimentation, or inability to refuse unsafe treatment. Lowering one’s voice as a context-specific courtesy does not by itself diminish agency; neither does consulting adults about consequential choices. Risk arises when quietness becomes a global index of goodness, ordinary initiative requires permission, or protest against adult action is sanctioned. The child may then have a judgment and motivation but lack a socially protected route to act on them.

This dimension connects participation rights to everyday pedagogy. Children need accessible opportunities to express views, receive a response, and seek help without forfeiting belonging (Committee on the Rights of the Child, 2009). Dialogic classroom research suggests that shared reasoning requires norms of constructive challenge and genuine uptake rather than mere turn allocation (Mercer et al., 2019). In *Di Zi Gui* teaching, the relevant evidence would include who asks questions, who defines correctness, what happens after disagreement, whether children can name exceptions, and whether adult conduct is also open to evaluation.

### 6.4 An adultcentric feedback loop

A possible feedback loop is also hypothesized. Where interpretive authority is monopolized, a child may reduce questioning, conceal disagreement, or seek permission for manageable decisions. An adult could read this situated response as evidence of incapacity and use that interpretation to justify tighter control. The resulting enactment might then provide still fewer opportunities to exercise judgment. However, the same visible dependence could reflect unfamiliarity, insecurity, strategic cooperation, or a need for support. Establishing the loop would require repeated observations of the child’s reception, the adult’s interpretation, and subsequent enactment; it cannot be inferred from silence or one episode of compliance.

A further untested possibility concerns adults themselves. A parent or teacher who understands authority as requiring certainty may find it difficult to acknowledge error or accept correction. Children would then encounter fewer examples of responsible fallibility. This extension would require adult accounts and repeated interactional evidence; it is not an observed consequence of the text or an additional established mechanism.

Children’s reinterpretation, strategic compliance, selective appropriation, or resistance could redirect this sequence. Dialogical inquiry, reciprocal adult modeling, and consequential participation are proposed moderators because they might change how reasons and dissent are received. None is an established interruption of the loop. Figure 1 distinguishes the conceptual sequence from the statistical roles specified in P1–P5; its arrows are hypotheses, not observed effects. The empirical issue is whether changes in enactment and reception predict subsequent outcomes, including cases where the expected risk does not appear.

**FIGURE 1:**
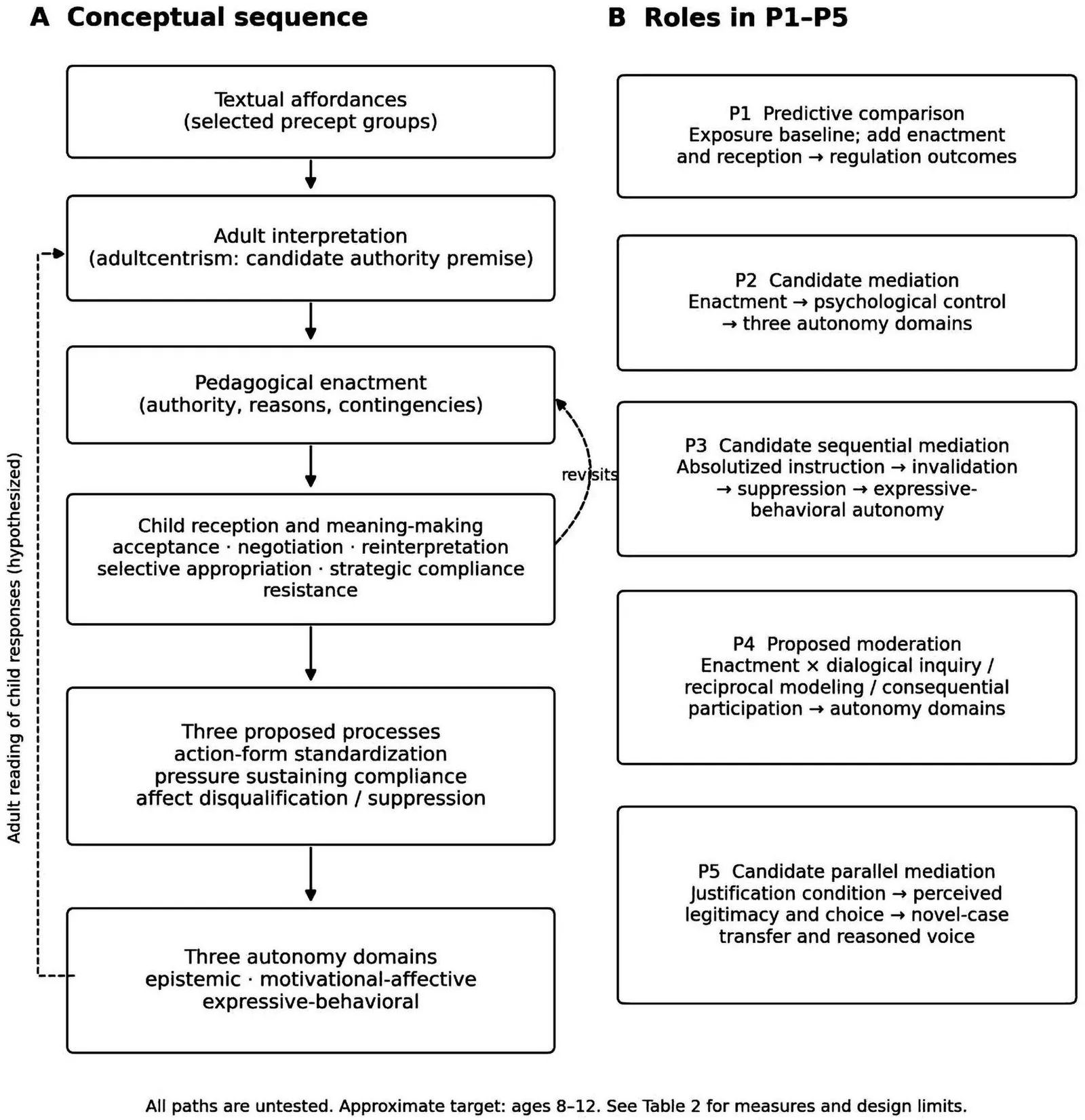
Conceptual sequence and statistical roles in the proposed model. **(A)** Presents hypothesized relations, not measured effects. Reception may also reshape subsequent enactment; the return arrow does not imply an estimated feedback coefficient. The process-moderator box concerns enactment–autonomy associations (P4), not a demonstrated direct benefit. **(B)** Distinguishes predictive comparison (P1), candidate mediators (P2, P3, P5), and moderators (P4); the detailed variable roles are specified in Table 2. The population focus is approximately 8–12 years, subject to developmental and cultural validation.

**TABLE 2:** Testable propositions and suggested future measures.

ID	Predictor / comparison	Mediator / moderator	Outcome	Operational distinction	Design / challenge
P1	Exposure + covariates + baseline regulation; then add enactment and episode-specific reception	None specified; reception is an added predictor	Subsequent external, introjected, and identified regulation	Reception codes describe interpretation/response; separate regulation measures assess outcome	Prospective nested models with penalization or held-out prediction; no incremental gain challenges claim
P2	Authoritarian enactment (T1); rationale-based structure as contrast	Perceived psychological control (T2), candidate mediator	Three autonomy domains (T3), controlling prior levels	Observed adult behavior, perceived intrusion, and autonomy use nonoverlapping indicators	Prospective temporal design; absent indirect path or reversed ordering challenges claim
P3	Absolutized filial instruction (T1)	Perceived emotion invalidation (T2), then expressive suppression (T3)	Expressive-behavioral autonomy (T4): later dissent, disclosure, help-seeking	Adult response to affect ≠ child concealment ≠ subsequent opportunity/action	Prospective observation; missing sequence challenges account; do not induce harmful teaching
P4	Authoritarian enactment	Dialogical inquiry, reciprocal modeling, consequential participation: distinct moderators	Three autonomy domains	Trace child contribution → adult response → change in interpretation/practice; talk counts insufficient	Prespecified enactment-by-process interactions; null/reversed interaction challenges prediction
P5	Adult-status versus mutually applicable reason justification; same precept	Perceived legitimacy and choice: parallel candidate mediators	Transfer to a novel case and willingness to voice a reasoned objection	Mediators assessed after justification, before separate transfer/voice tasks	Safe randomized vignette/micro-comparison; equal outcomes or unsupported indirect paths challenge claim

P1 treats regulation quality only as an outcome; reception is an added predictor, not a synonym for the regulation score. P5 treats perceived legitimacy and choice only as parallel mediators, and transfer and reasoned voice as outcomes. T1–T4 denote proposed measurement occasions, not observations collected for this article. All pathways and interactions remain hypotheses. Measures require linguistic, developmental, and cultural validation; temporal separation and statistical mediation alone do not establish causation.

## 7 Dialogical reconstruction through philosophy for children

### 7.1 Critical inheritance

The proposed reconstruction neither requires compulsory literalism nor wholesale rejection. It treats *Di Zi Gui* as inherited material to be interpreted through care, honesty, reciprocity, and responsibility, including tensions among its own precepts. Philosophy for Children (P4C) supplies a possible community-of-inquiry format, not a culturally external replacement assumed to be superior. Cam describes the educational aim as “good judgment through collaborative inquiry” (2014, p. 1209). Whether such inquiry changes authority relations in a particular setting remains to be investigated.

This stance is consistent with tensions inside the text. The injunctions to care for all people and to test one’s conduct through reciprocity offer broad moral criteria (Li, 2005, pp. 8–9). The contrast between force and reasons questions outward submission as a sufficient educational goal, and the directive to record doubts and seek clarification recognizes inquiry as part of learning (pp. 9–10). Reconstruction does not use these passages to sanitize the severe ones. It lets competing lines become an object of moral inquiry: whether perseverance in remonstrance can be separated from tolerating violence; whether respect entails agreement; and whether duties assigned to the young should also constrain adults.

### 7.2 A six-step dialogical model

Philosophy for Children provides a practical grammar for this reconstruction through a community of inquiry (Cam, 2014). Six linked moves can organize a lesson. First, historical contextualization identifies the primer’s genre, household assumptions, linguistic compression, and distance from contemporary life. The point is not to excuse every rule but to prevent present application from masquerading as timeless meaning. Second, child-generated questioning gives participants time to identify what genuinely puzzles or troubles them. The adult may clarify vocabulary without replacing children’s questions with a predetermined moral objective.

Third, ethical-value identification asks what concern a precept might protect: safety, attention, trust, care, fairness, or social coordination. Fourth, cases and counterexamples test its scope: What if a parent calls during an emergency? What if an instruction harms another person? What if a child’s delay has a good reason? Fifth, reciprocal reason-giving asks children and adults to justify claims using mutually examinable considerations rather than status. The proposed facilitation draws on critical, constructive engagement and uptake of children’s contributions in dialogic teaching (Mercer et al., 2019), without assuming that this format has already produced the intended change in authority.

Sixth, participants formulate provisional, co-authored norms. A reconstructed rule should identify its value, who it applies to, relevant exceptions, and how disagreement will be handled. “Family members should respond to one another when possible and explain necessary delays” retains attentiveness without making adult summons absolute. Provisionality does not mean that every safety rule is optional. It means that even a firm rule has a public rationale, a defined scope, and a pathway for reconsideration as competence and conditions change.

The six moves are not a validated *Di Zi Gui* intervention protocol. They operationalize theoretical moderators: contextual interpretation, child participation, reciprocal adult modeling, and public reason. Their effectiveness, developmental suitability, and fidelity would require direct study.

### 7.3 Transforming selected precepts

Consider “When parents instruct, listen respectfully; when parents admonish, accept the admonition compliantly” (Li, 2005, p. 3, author’s translation). An adultcentric lesson equates listening with agreement. A dialogical lesson asks whether attentive listening can coexist with factual correction, whether parents can misjudge a situation, and what a respectful disagreement sounds like. The provisional norm might require everyone first to understand the other’s account, then respond with reasons. Respect becomes a procedure for disagreement rather than immunity from it.

The instruction to notify others when leaving and returning provides a different test case (T04; Li, 2005, p. 3). A group could examine which decisions involve safety or shared consequences and which permit greater discretion. A proposed graded map–adult decision, joint decision, child decision–would require reasons grounded in risk and developing competence. The exercise would not itself demonstrate that discretion or autonomy increased; that claim would require evidence about subsequent practice and the child’s understanding.

The injunction to remain without resentment even when struck requires a non-negotiable rights boundary. Inquiry can ask whether enduring harm proves care, whether anger can reveal injustice, and whom a child may contact for help. The reconstructed norm should state that no filial obligation requires accepting violence; a child may refuse, leave danger, disclose the event, and seek protection. Here dialogue does not suspend adult responsibility until consensus is reached. The adult facilitator must establish safety while still allowing participants to examine how an inherited formulation came to conflate endurance with virtue.

“It is better to speak little than too much” can be tested against cases of gossip, interruption, reporting harm, correcting misinformation, and defending someone excluded (Li, 2005, p. 7, author’s translation). Restraint may serve truthfulness in the first cases but undermine responsibility in the latter. The reason-over-force and inquiry verses can then serve as internal warrants for revision. The outcome is not a new universal slogan but a norm such as: speak truthfully and proportionately, and do not let conventions of quietness prevent necessary questions, disagreement, or help-seeking.

### 7.4 Responsible and fallible adult authority

Dialogical classroom form and redistribution of interpretive authority must be distinguished. Children may take turns, discuss a verse, and ask questions while the adult still predetermines the permissible conclusion. Mercer et al. (2019) describe dialogue in terms of critical, constructive engagement and jointly constructed understanding; the relevant issue here is what follows a contribution, not the quantity of talk. Candidate evidence of consequential participation includes a child’s reason changing an interpretation, exception, procedure, or provisional norm, and an adult explaining why a proposal is accepted or rejected without penalizing dissent. Counts of turns, open questions, or P4C sessions are exposure or format measures, not sufficient measures of that process.

Philosophy for Children is therefore only a proposed moderator. Studies should track episodes from contribution through adult response to subsequent practice, compare those records with children’s accounts of whether they could disagree safely, and examine whether publicly revised norms are actually followed. Adults retain protective duties and may reject unsafe proposals with reasons; participation does not mean automatic child decision-making. A null interaction, ritual discussion without uptake, or unequal opportunities for less verbally confident children would challenge the moderation account. No direct trial in the present evidence base establishes that P4C neutralizes autonomy-related risks in *Di Zi Gui* instruction.

The shift also echoes Bai’s, 2014 call for “equal dialogue and emotional communication” in the contemporary use of the primer (2014, p. 37, author’s translation). Equality here does not erase adults’ special duties; it means that neither age nor instructional role makes a claim immune from reasons or an emotional report morally inadmissible.

## 8 Discussion and testable propositions

### 8.1 Contributions and boundary conditions

The contribution is a conditional research framework, not a finding that the primer diminishes autonomy. It complements Luo and Yang (2026) and Santana (2026) by linking a specified text to enactment, child reception, and differentiated psychological hypotheses. The core theoretical contrast is between nonreciprocal interpretive authority and the conditions under which commitments can be understood and endorsed. Psychological control and emotion invalidation identify candidate processes; they do not convert textual analysis into outcome evidence. Behavioral structure, active filial identification, and prosocial benefit remain genuine alternatives rather than concessions added after a predetermined critical conclusion (Deci and Ryan, 2000; Shek and Law, 2019).

The primary comparison must be tested directly. An exposure-only model should include contact frequency, duration, and setting, alongside prespecified background covariates and baseline outcomes. An expanded model should add independently assessed enactment, and then reception of specific instructional episodes. Incremental explanatory value should be evaluated with out-of-sample prediction or an appropriately penalized nested-model comparison, not assumed from adding predictors. Such improvement would support the comparative hypothesis, not prove causation; reverse influence, family selection, instructor characteristics, and shared-method variance could remain. Reliable failure of enactment and reception to add information would challenge the primary hypothesis.

Developmental and contextual alternatives require design rather than generic disclaimers. The initial target is approximately 8–12 years: exact age, language comprehension, prior instruction, family relationships, and school climate should be measured, and age interactions prespecified where feasible. Evidence from adolescents or adult recollections supplies theoretical background, not measurement validity for this group. Younger children may need observational and play-based elicitation; adolescent applications would need to examine changing peer, identity, and authority relations. These extensions are not assumed to follow one linear age gradient. Nor should demanding filial commitment be classified as controlled before children’s own reasons are examined.

### 8.2 Five propositions

The following propositions are untested. They prioritize children aged approximately 8–12 and require age- and context-appropriate measures. A predictor, mediator, moderator, and outcome are distinct roles: temporal language specifies a future design, not a causal sequence demonstrated by this article. Table 2 uses the same roles as the prose.

Proposition 1. Enactment conditions and episode-specific reception are expected to improve prediction of subsequent external, introjected, and identified regulation beyond an exposure-only baseline and baseline regulation. Reception is coded as acceptance, negotiation, reinterpretation, selective appropriation, strategic compliance, or resistance, using evidence separate from the outcome scales. A directional subprediction is that mandatory, decontextualized enactment will be more positively associated with external and introjected than with identified regulation, relative to rationale-rich instruction. P1 specifies added predictors and regulation outcomes, not regulation quality as its own mediator. No incremental predictive gain, or no differentiation among regulation outcomes, would weaken the corresponding claims.

Proposition 2. Authoritarian enactment is expected to predict lower subsequent epistemic, motivational-affective, and expressive-behavioral autonomy through perceived psychological control. The proposed design separates observed enactment at T1, child-reported psychological control at T2, and autonomy at T3, adjusting for prior mediator and outcome levels. Rationale-based behavioral structure is a contrasting predictor, not another name for control. An absent indirect association, reversed temporal ordering, or indistinguishability of structure and control would challenge the account. Temporal separation alone does not eliminate unmeasured confounding.

Proposition 3. Absolutized filial instruction at T1 is expected to predict reduced expressive-behavioral autonomy at T4 through perceived emotion invalidation at T2 and expressive suppression at T3. Invalidation concerns the adult’s response to affect; suppression concerns the child’s subsequent concealment; the outcome concerns later dissent, disclosure, and help-seeking opportunities or action. These must use nonoverlapping indicators. A sequence in which emotion remains acknowledged, concealment does not follow, or later expression is unaffected would challenge this pathway. No coercive or harmful enactment should be experimentally induced to test it.

Proposition 4. Dialogical inquiry, reciprocal adult modeling, and consequential child participation are proposed moderators of the association between authoritarian enactment and the three autonomy domains. Each is to be measured as a distinct process rather than collapsed into discussion frequency. Tests should examine prespecified enactment-by-process interactions and whether children’s contributions actually change interpretation or practice. A weaker negative association under stronger process conditions would support the moderation prediction; a null or reversed interaction would not. An intervention’s total effect and a statistical moderation effect are different estimands and should not be treated as interchangeable evidence.

Proposition 5. In an ethically appropriate randomized vignette or brief instructional comparison holding the precept constant, adult-status justification is expected to be associated with lower subsequent transfer of its moral value to a novel case and less willingness to voice a reasoned objection than mutually applicable reasons. Perceived legitimacy and perceived choice are proposed parallel mediators, measured after the justification and before the outcome tasks. They are not additional outcomes in P5. Randomization can support a comparison of justification conditions, but mediation still requires assumptions about mediator–outcome confounding. Equivalent transfer and voice across conditions, or unsupported indirect paths, would challenge the specified predictions.

For all five propositions, construct development precedes pathway testing. Interviews and cognitive testing should establish how children understand items; discriminant and developmental validity should be examined before pooling ages or settings. Exposure, adult behavior, reception, and outcomes should not all be inferred from one questionnaire. Adultcentrism scales assess adult orientations and cannot substitute for children’s accounts. Observations should distinguish action-form rigidity, motivational pressure, and emotional invalidation as specified in “Section 5 Three proposed adultcentric psychological mechanisms.” Cross-sectional indirect associations cannot establish temporal mediation, and exploratory models must be identified as such. Proposed tests should also allow null findings and rival explanations without reclassifying every response as hidden evidence of adultcentrism.

### 8.3 Limitations and research agenda

The article contains no original participant data, children’s interviews, or direct observations of current enactment. It cannot estimate prevalence, effect size, or causal impact, and none of its three mechanisms is empirically established for *Di Zi Gui* instruction. Its 34-group purposive selection privileges one modern working edition, is not an exhaustive coding of that edition, and does not collate early witnesses. The revision-stage ledger improves inspectability but cannot supply an undocumented coding history or inter-rater reliability. Available implementation sources do not isolate text, pedagogy, instructor behavior, or participant selection; Yao and Wong (2021) remains abstract-based. Described enactment scenarios are theoretical possibilities, not claims about typical current practice. The approximately 8–12-year focus is a proposed research scope, not a demonstrated zone of effects. Variation by age, gender, disability, region, socioeconomic position, religion, and educational setting remains unresolved, as does the cultural and developmental validity of the outcome constructs.

Future work should begin with situated observation and child-centered interviews, including counter-cases of endorsed obligation, negotiation, and supportive structure. Measures may draw on Zhu et al. (2023), but their adolescent scale is not automatically valid in middle childhood or for classroom authority. Subsequent prospective studies can test the exposure–enactment–reception comparison and the differentiated pathways after construct validation. The P4C proposal requires evidence of consequential uptake, not merely a discussion format. These steps would test the model rather than retrospectively confirm a conclusion already attributed to the text.

## 9 Conclusion

*Di Zi Gui* provides material for a conditional psychological model, not evidence that its readers experience diminished autonomy. The present analysis distinguishes textual affordances, interpretation, enactment, reception, candidate mechanisms, and possible outcomes. It proposes that restrictions on action form, pressure sustaining conformity, and moral disqualification of affect may matter under specified conditions. Children’s meaning-making and the text’s resources for care, reciprocity, remonstrance, and inquiry also make other pathways conceivable.

The autonomy at issue is relational and culturally situated, compatible with endorsed filial obligations but not reducible to outward obedience. For the proposed middle-childhood focus, the testable question is whether enactment and reception improve prediction beyond exposure, and whether the differentiated processes survive discriminant, longitudinal, and contextual tests. P4C offers one possible moderator only where interpretive participation is consequential. Without child accounts and enactment observations, the model remains an agenda for empirical inquiry.

## Data Availability

The original contributions presented in this study are included in this article/Supplementary material, further inquiries can be directed to the corresponding author.

## References

[B1] Assor A. Roth G. Deci E. L. (2004). The emotional costs of parents’ conditional regard: A self-determination theory analysis. *J. Personal.* 72, 47–88. 10.1111/j.0022-3506.2004.00256.x 14686884

[B2] BaiY.-J. (2014). 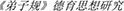 [*A Study of the Moral-Education Thought in Di Zi Gui*]. Master’s thesis, Harbin: Northeast Forestry University.

[B3] Biswas T. Florio E. Caso L. Castelli I. Iacobino S. (2024). The Adultcentrism Scale: A potential contributor to advancing children’s participation rights in Nordic contexts. *Soc. Sci.* 13:238. 10.3390/socsci13050238

[B4] Cam P. (2014). Philosophy for Children, values education and the inquiring society. *Educ. Philos. Theory* 46, 1203–1211. 10.1080/00131857.2013.771443

[B5] Chirkov V. Ryan R. M. Kim Y. Kaplan U. (2003). Differentiating autonomy from individualism and independence: A self-determination theory perspective on internalization of cultural orientations and well-being. *J. Personal. Soc. Psychol.* 84, 97–110. 10.1037/0022-3514.84.1.97 12518973

[B6] Committee on the Rights of the Child (2009). *General Comment No. 12: The Right of the Child to be Heard (CRC/C/GC/12).* Geneva: United Nations.

[B7] Deci E. L. Ryan R. M. (2000). The “what” and “why” of goal pursuits: Human needs and the self-determination of behavior. *Psychol. Inquiry* 11, 227–268. 10.1207/S15327965PLI1104_01

[B8] Florio E. Caso L. Castelli I. (2020a). The Adultcentrism Scale in the educational relationship: Instrument development and preliminary validation. *New Ideas Psychol.* 57:100762. 10.1016/j.newideapsych.2019.100762

[B9] Florio E. Caso L. Castelli I. (2020b). The black pedagogy scale: A new task to explore educational practices for children’s well-being. *Eur. J. Psychol.* 16, 331–351. 10.5964/ejop.v16i2.1876 33680186 PMC7913014

[B10] Florio E. Caso L. Castelli I. (2022). Detrimental educational practices deemed as culturally acceptable: Adultcentrism and Black Pedagogy in Italian primary schools. *Soc. Psychol. Educ.* 25, 313–347. 10.1007/s11218-021-09676-1

[B11] Gross J. J. (1998). Antecedent- and response-focused emotion regulation: Divergent consequences for experience, expression, and physiology. *J. Personal. Soc. Psychol.* 74, 224–237. 10.1037/0022-3514.74.1.224 9457784

[B12] HuangC. (2011). 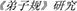 [*A Study of Di Zi Gui*]. Master’s thesis, Shanghai: Shanghai Normal University.

[B13] Kağıtçıbaşı Ç (2005). Autonomy and relatedness in cultural context: Implications for self and family. *J. Cross-Cult. Psychol.* 36, 403–422. 10.1177/0022022105275959

[B14] LiY. (2005). “ 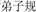 *Di Zi Gui*”. in 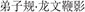 [*Di Zi Gui and Longwen Bianying*], ed. 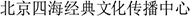 [Beijing Sihai Classics Cultural Communication Center]. Beijing: Zhonghua Book Company.

[B15] LinY.-T. (2018). 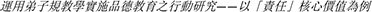 [*An action study of character education through Di Zi Gui instruction: The core value of responsibility*]. Master’s thesis, Hsinchu: National Tsing Hua University.

[B16] Luo Y. Yang X. (2026). The ontological inversion: Transcending adultcentrism in the concept of development for the critical deconstruction of bleak pedagogy. *Front. Psychol.* 17:1794220. 10.3389/fpsyg.2026.1794220 41937850 PMC13044036

[B17] Mercer N. Hennessy S. Warwick P. (2019). Dialogue, thinking together and digital technology in the classroom: Some educational implications of a continuing line of inquiry. *Intern. J. Educ. Res.* 97, 187–199. 10.1016/j.ijer.2017.08.007

[B18] Morris A. S. Silk J. S. Steinberg L. Myers S. S. Robinson L. R. (2007). The role of the family context in the development of emotion regulation. *Soc. Dev.* 16, 361–388. 10.1111/j.1467-9507.2007.00389.x 19756175 PMC2743505

[B19] Petr C. G. (1992). Adultcentrism in practice with children. *Fam. Soc.* 73, 408–416. 10.1177/104438949207300703

[B20] QinQ. (2018). 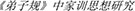 [*A Study of Family-Instruction thought in Di Zi Gui*]. Master’s thesis, Nanning: Guangxi Minzu University.

[B21] Roth G. Assor A. (2010). Parental conditional regard as a predictor of deficiencies in young children’s capacities to respond to sad feelings. *Infant Child Dev.* 19, 465–477. 10.1002/icd.676

[B22] Roth G. Assor A. Niemiec C. P. Ryan R. M. Deci E. L. (2009). The emotional and academic consequences of parental conditional regard: Comparing conditional positive regard, conditional negative regard, and autonomy support as parenting practices. *Dev. Psychol.* 45, 1119–1142. 10.1037/a0015272 19586183

[B23] Santana D. B. (2026). Emotional violence by teachers toward students: The adultcentric bias hypothesis. *Front. Psychol.* 17:1849830. 10.3389/fpsyg.2026.1849830 42382633 PMC13314923

[B24] Shek D. T. L. Law M. Y. M. (2019). “*Di Zi Gui*” (Standards for being a good student and child): Implications for children and youth development and parenting. *Intern. J. Child Adolescent Health* 12, 403–418.

[B25] ShenY.-Y. (2018). 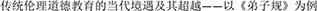 [The contemporary situation and transcendence of traditional moral education: Taking *Di Zi Gui* as an example]. *Teacher Educ. Forum* 31, 8–11.

[B26] United Nations (1989). *Convention on the Rights of the Child.* New York, NY: United Nations.

[B27] Yao Z. Wong L. (2021). The effect of the Dizi Gui intervention on peer relationships and teacher–student relationships: The mediating role of prosocial behavior. *J. Moral Educ.* 50, 384–400. 10.1080/03057240.2020.1722080

[B28] ZhangY.-X. (2019). “ 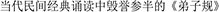 [The Controversial Di Zi Gui in Contemporary Popular Classics Recitation],” in 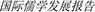 (2016–2017) *[Proceedings of the International Confucianism Development Report 2016–2017]*, (Jinan: Shandong Friendship Publishing House), 141–165.

[B29] Zhu Y. Dou K. Pan Y. (2023). Developing and validating a multidimensional Chinese parental psychological control scale. *Front. Psychol.* 14:1116625. 10.3389/fpsyg.2023.1116625 37034931 PMC10077889

