# Supplementary material for: Adultcentrism in the moral instruction of Di Zi Gui: proposed psychological mechanisms and implications for children’s autonomy
Source: Front Psychol. 2026 Oct 16;17:1989993. doi: 10.3389/fpsyg.2026.1989993 (PMC13719386; doi:10.3389/fpsyg.2026.1989993)
Supplement: Supplementary file 1 [file Supplementary_file_1.docx]

Supplementary Material S1

Selected-precept ledger, analytical criteria, and source-check record
Revised 21 September 2026

# S1. Purpose and evidentiary status

This supplement makes the selected primary-text inventory and conditional interpretations inspectable. It contains no participant observations, coding frequencies, effect estimates, or validated mechanism scores.

The retained project workbook contains 34 selected precept groups, T01–T34. Table 1 illustrates 10 groups. Thirty-four is not an exhaustive segmentation of the edition: the full-text denominator and the chronology of selecting and coding all possible units are not documented. This publication-facing ledger is a revision-stage reconstruction from that workbook, the supplied working source, and the prior manuscript. It is not presented as a contemporaneous independent-coder record.

All 34 selected passages and their printed-page locators were checked against the clear scan of the 2005 working edition on 21 September 2026. The title and copyright pages establish the volume title, editor, publisher, and edition. This check resolves the earlier qualifications for T03, T05, T17, and T22. Source verification concerns wording and location; it does not validate the proposed psychological mechanisms. This is a check against one modern edition, not a collation of early witnesses.

Selection includes conduct, authority, correction, and emotion prescriptions and counter-cases involving care, reciprocity, reasons, remonstrance, and inquiry. No independent double-coding procedure, author-specific coding assignment, formal adjudication, or agreement statistic is documented in the available audit trail. None has been invented for this revision. The authors’ actual division of interpretive work must be reported only if they can verify it.

# S2. Source, translation, and coding conventions

All T locators refer to Li Yuxiu’s *Di Zi Gui* in the 2005 Zhonghua Book Company volume 弟子规·龙文鞭影, edited by 北京四海经典文化传播中心 [Beijing Sihai Classics Cultural Communication Center]. The title page and copyright page occur at PDF images 3 and 4; the latter states first edition and first printing, July 2005. The complete primer occupies printed pp. 3–11, corresponding to PDF images 13–21. The selected passages occupy printed pp. 3–10 (PDF images 13–20). PDF image numbers are one-based and include the covers and front matter. The uploaded filename is not the bibliographic title. T identifiers refer to analytical groups, not the edition’s circled recitation numbers.

Chinese punctuation is normalized for readability; it is not attributed to the Qing-era author. English renderings are the authors’ translations and distinguish description from interpretation: 顺承 is rendered as compliant acceptance, not absolute obedience; 挞无怨 as absence of resentment if struck, not permission to use violence; 理 and 心不然 are not silently translated as modern psychological autonomy. T11 retains the phrase about following with cries and tears. T03 follows the scan’s 清, and T22 retains its 则; these edition-specific readings are recorded without claiming that they establish the earliest text.

Each entry records nine analytic dimensions in paired fields: value and directive form; authority and reciprocity; sanction and emotional rule; reasons and contextual qualification; then a conditional mechanism mapping. A missing reason, sanction, or exception means it is not explicit in that precept group, not that it is absent from the wider text or actual teaching. Neighboring remonstrance passages and cross-text counter-cases are retained. Multiple mappings require distinct enactment criteria; an ambiguous group is not forced into one category.

Behavioral standardization requires privileging a prescribed action form despite a reasoned alternative. Compliance reinforcement requires an observed or perceived contingency sustaining conformity through pressure, sanction, approval, or self-worth. Emotional suppression through moral absolutization requires moral disqualification of an affect and evidence of concealment or inhibition. These are proposed criteria for future inquiry, not properties proven by the verses. All mappings must be tested against children’s reception and constructive alternatives.

# S3. The 34 selected precept groups

Paired fields below implement the nine dimensions described in Section 4.2. Every proposed mechanism requires evidence about enactment and reception that this article does not contain. Source status concerns transcription and location, not the truth of the psychological mapping.

## T01 · Li (2005), p. 3

父母呼，应勿缓；父母命，行勿懒

**Translation:** When parents call, respond without delay; when parents give an instruction, do not be lax in carrying it out.

**Value / form:** Attentiveness; paired commands. Authority / reciprocity: Parent directs, child responds; reciprocity unstated. Sanction / affect: No explicit penalty or feeling rule. Reasons / qualification: Rationale and exceptions unstated.

**Conditional mapping / counter-reading:** Action-form rigidity only if reasoned delays are rejected; motivational reinforcement additionally requires a contingency. Prompt response can instead express endorsed care.

Scan checked: printed p. 3; PDF image 13 (21 September 2026).

## T02 · Li (2005), p. 3

父母教，须敬听；父母责，须顺承

**Translation:** When parents instruct, listen respectfully; when parents admonish, accept the admonition compliantly.

**Value / form:** Receptivity; commands. Authority / reciprocity: Parent teaches or corrects; reciprocal correction unstated here. Sanction / affect: Respectful/acquiescent reception; no specified penalty. Reasons / qualification: Reasons and exceptions unstated.

**Conditional mapping / counter-reading:** Compliance reinforcement requires pressure on approval, worth, or belonging. Respectful attention need not mean agreement; remonstrance elsewhere is a counter-resource.

Scan checked: printed p. 3; PDF image 13 (21 September 2026).

## T03 · Li (2005), p. 3

冬则温，夏则清；晨则省，昏则定

**Translation:** In winter provide warmth, in summer coolness; inquire in the morning and attend in the evening.

**Value / form:** Care; seasonal/daily instructions. Authority / reciprocity: Child attends to parents; reciprocity unstated. Sanction / affect: No explicit penalty or prohibited feeling. Reasons / qualification: Time and season qualify conduct.

Conditional mapping / counter-reading: Rigid routine is only a possible enactment. Care adapted to need is a constructive reading. The contextual translation of 清 as coolness does not silently replace the printed graph.

Scan checked: printed p. 3; PDF image 13. The scan prints 清, not 凊. The transcription now follows the working edition; “coolness” conveys the seasonal contrast in context. This resolves the earlier image/text-layer uncertainty without claiming a collation of other editions.

## T04 · Li (2005), p. 3

出必告，反必面；居有常，业无变

**Translation:** When going out, inform them; on returning, present yourself. Keep a regular place of residence and do not change your occupation or activity.

**Value / form:** Notification and constancy; commands. Authority / reciprocity: Child reports; reciprocity unstated. Sanction / affect: No explicit sanction or affect rule. Reasons / qualification: Going/returning are specified contexts; rationale unstated.

**Conditional mapping / counter-reading:** Standardization requires rejection of reasonable discretion. Notification may serve safety and coordination. The scope of 業 is interpretive, not a modern career rule inferred as fact.

Scan checked: printed p. 3; PDF image 13 (21 September 2026).

## T05 · Li (2005), p. 3

事虽小，勿擅为；苟擅为，子道亏

**Translation:** Though a matter is small, do not act on your own; if you do, your filial role is compromised.

**Value / form:** Caution/filial role; prohibition with consequence. Authority / reciprocity: Child discretion limited; reciprocity unstated. Sanction / affect: Filial-role deficiency; no specific feeling commanded. Reasons / qualification: Smallness does not exempt action.

Conditional mapping / counter-reading: Standardization would require treating a restriction on unilateral action as excluding justified initiative in enactment. A restriction could instead concern shared consequences. The wording alone establishes neither an unlimited prohibition nor an autonomy outcome.

Scan checked: printed p. 3; PDF image 13. The complete wording 事虽小，勿擅为；苟擅为，子道亏 is legible. The earlier transcription qualification is resolved. This entry remains in S1; Table 1 retains its existing 10 examples.

## T06 · Li (2005), p. 3

物虽小，勿私藏；苟私藏，亲心伤

**Translation:** Though an object is small, do not privately keep it; if you do, your parents will be saddened.

**Value / form:** Trust; prohibition. Authority / reciprocity: Child conduct referred to parents; reciprocity unstated. Sanction / affect: Parents’ hurt is named; child feeling not directly prescribed. Reasons / qualification: Small value does not excuse concealment.

**Conditional mapping / counter-reading:** Compliance reinforcement requires an enacted guilt/approval contingency. The line can instead counsel honesty about shared property; private keeping is not automatically theft.

Scan checked: printed p. 3; PDF image 13 (21 September 2026).

## T07 · Li (2005), p. 4

亲所好，力为具；亲所恶，谨为去

**Translation:** What parents like, strive to provide; what parents dislike, carefully remove.

**Value / form:** Attentive care; commands. Authority / reciprocity: Parental preferences orient child conduct; reciprocity unstated. Sanction / affect: No explicit sanction or child-feeling rule. Reasons / qualification: Parental liking/disliking is the criterion.

**Conditional mapping / counter-reading:** Standardization is possible if preferences become unquestionable duties. Voluntary care remains an alternative; no psychological outcome is in the wording.

Scan checked: printed p. 4; PDF image 14 (21 September 2026).

## T08 · Li (2005), p. 4

身有伤，贻亲忧；德有伤，贻亲羞

**Translation:** If the body is injured, one brings worry to one’s parents; if one’s moral conduct is harmed, one brings shame to one’s parents.

**Value / form:** Care/responsibility; relational consequences. Authority / reciprocity: Child’s condition linked to parents; reciprocity unstated. Sanction / affect: Parental worry and shame are explicit. Reasons / qualification: Relational consequences offer a rationale; exceptions unstated.

**Conditional mapping / counter-reading:** Compliance reinforcement requires using worry/shame to pressure worth or belonging. Considering effects on others need not be psychological control.

Scan checked: printed p. 4; PDF image 14 (21 September 2026).

## T09 · Li (2005), p. 4

亲爱我，孝何难？亲恶我，孝方贤

**Translation:** If parents love me, how is filiality difficult? If parents dislike me, only then is filiality worthy.

**Value / form:** Filial constancy; evaluative contrast. Authority / reciprocity: Duty persists under parental dislike; reciprocity not required here. Sanction / affect: Parental love/dislike; child feeling not explicitly forbidden. Reasons / qualification: Contrast evaluates difficult filial commitment.

**Conditional mapping / counter-reading:** Affective suppression requires teaching that hurt or anger is illegitimate. Perseverance and self-command are possible alternative readings.

Scan checked: printed p. 4; PDF image 14 (21 September 2026).

## T10 · Li (2005), p. 4

亲有过，谏使更；怡吾色，柔吾声

**Translation:** If parents are at fault, remonstrate so that they change; make our expression pleasant and our voice gentle.

**Value / form:** Correction with respect; instruction. Authority / reciprocity: Parents can err; child can remonstrate. Sanction / affect: Facial/vocal manner specified; no explicit sanction. Reasons / qualification: Parental wrongdoing warrants correction.

**Conditional mapping / counter-reading:** Counter-case to interpretive monopoly. A prescribed manner could still be rigidly enforced, but the permission to remonstrate must not be erased.

Scan checked: printed p. 4; PDF image 14 (21 September 2026).

## T11 · Li (2005), p. 4

谏不入，悦复谏；号泣随，挞无怨

**Translation:** If remonstrance is not accepted, remonstrate again when they are pleased; follow them with cries and tears; if struck, harbor no resentment.

**Value / form:** Persistence; counsel and affective injunction. Authority / reciprocity: Child may correct; adult retaliation not explicitly prohibited here. Sanction / affect: Being struck and absence of resentment are explicit. Reasons / qualification: Repeated remonstrance qualified by receptiveness.

**Conditional mapping / counter-reading:** Affective suppression requires moral disqualification and concealment in enactment/reception. Read with T10; the translation does not make the passage an adult authorization to strike.

Scan checked: printed p. 4; PDF image 14 (21 September 2026).

## T12 · Li (2005), p. 4

丧三年，常悲咽；居处变，酒肉绝

**Translation:** Mourn for three years, often sobbing with grief; change one’s living arrangements and abstain from wine and meat.

**Value / form:** Mourning; ritual instructions. Authority / reciprocity: Bereaved learner; authority not explicit in these lines. Sanction / affect: Grief and abstinence prescribed; penalty unstated. Reasons / qualification: Bereavement context limits application.

**Conditional mapping / counter-reading:** Suppression hypothesis applies only if other feelings are forbidden or a display is coerced. A meaningful mourning practice is an alternative.

Scan checked: printed p. 4; PDF image 14 (21 September 2026).

## T13 · Li (2005), p. 4

兄道友，弟道恭；兄弟睦，孝在中

**Translation:** The elder brother’s way is friendliness; the younger brother’s way is respect. When siblings live harmoniously, filiality is present within it.

**Value / form:** Sibling harmony; paired duties. Authority / reciprocity: Different roles; elder as well as younger has an obligation. Sanction / affect: No explicit sanction or prohibited feeling. Reasons / qualification: Harmony links duties to filiality.

**Conditional mapping / counter-reading:** Counter-resource to exclusively unilateral obligation. Role differentiation does not by itself establish equal power or an autonomy outcome.

Scan checked: printed p. 4; PDF image 14 (21 September 2026).

## T14 · Li (2005), p. 4

或饮食，或坐走；长者先，幼者后

**Translation:** Whether eating, drinking, sitting, or walking, elders go first and juniors follow.

**Value / form:** Courtesy/order; conduct rule. Authority / reciprocity: Priority follows age; reciprocity unstated. Sanction / affect: No explicit sanction or feeling rule. Reasons / qualification: Enumerated situations; exceptions unstated.

**Conditional mapping / counter-reading:** Standardization requires excluding justified alternatives concerning need, disability, urgency, or hosting. Contextual courtesy is not itself a deficit.

Scan checked: printed p. 4; PDF image 14 (21 September 2026).

## T15 · Li (2005), p. 5

称尊长，勿呼名；对尊长，勿见能

**Translation:** When addressing elders, do not call them by name; before elders, do not display your abilities.

**Value / form:** Deference/modesty; prohibitions. Authority / reciprocity: Junior before senior; reciprocity unstated. Sanction / affect: No specified penalty; restraint in self-display. Reasons / qualification: Elder-facing context; rationale unstated.

**Conditional mapping / counter-reading:** Standardization requires treating one display form as the only moral option. Modesty conventions need not forbid reasoned contribution or competence.

Scan checked: printed p. 5; PDF image 15 (21 September 2026).

## T16 · Li (2005), p. 5

路遇长，疾趋揖；长无言，退恭立。骑下马，乘下车；过犹待，百步余

**Translation:** Meeting an elder on the road, approach promptly and bow; if the elder does not speak, withdraw and stand respectfully. Dismount from a horse or alight from a carriage; after the elder passes, wait until they are more than a hundred paces away.

**Value / form:** Courtesy; sequential instructions. Authority / reciprocity: Junior accommodates senior; reciprocity unstated. Sanction / affect: Respectful bearing; penalty unstated. Reasons / qualification: Road encounter and travel specify context.

**Conditional mapping / counter-reading:** Possible rigid action script, not a present-day classroom observation. Historically specific ritual courtesy is a counter-reading.

Scan checked: printed p. 5; PDF image 15 (21 September 2026).

## T17 · Li (2005), p. 5

长者立，幼勿坐；长者坐，命乃坐

**Translation:** When an elder stands, a junior should not sit; when the elder sits, sit only when told.

**Value / form:** Deference; posture/permission rule. Authority / reciprocity: Elder controls seating permission; reciprocity unstated. Sanction / affect: No explicit sanction or affect rule. Reasons / qualification: Standing/seating context; exceptions unstated.

Conditional mapping / counter-reading: Standardization would require excluding justified seating needs. Context-sensitive courtesy is an alternative; the text alone does not establish how the rule is implemented or received.

Scan checked: printed p. 5; PDF image 15. The scan confirms 长者立，幼勿坐；长者坐，命乃坐 and resolves the earlier page/edition qualification.

## T18 · Li (2005), p. 5

尊长前，声要低；低不闻，却非宜

**Translation:** Before elders, one’s voice should be low; but so low that it cannot be heard is also inappropriate.

**Value / form:** Respect and audibility; command with qualification. Authority / reciprocity: Younger speaker adjusts; reciprocity unstated. Sanction / affect: Voice level specified, not an internal feeling. Reasons / qualification: Audibility explicitly limits the low-voice rule.

**Conditional mapping / counter-reading:** Standardization requires rigidity beyond this practical qualification; suppression additionally requires moralized invalidation. Low volume alone establishes neither.

Scan checked: printed p. 5; PDF image 15 (21 September 2026).

## T19 · Li (2005), p. 5

事诸父，如事父；事诸兄，如事兄

**Translation:** Serve those of one’s father’s generation as one serves one’s father; serve those of one’s elder brothers’ generation as one serves one’s elder brothers.

**Value / form:** Extended respect; analogical instruction. Authority / reciprocity: Senior generations receive service; reciprocity unstated. Sanction / affect: No explicit sanction or feeling rule. Reasons / qualification: Kin-like analogy gives scope.

**Conditional mapping / counter-reading:** Generalizing senior status into unquestionable authority is a possible enactment, not the only reading. Broad courtesy remains an alternative.

Scan checked: printed p. 5; PDF image 15 (21 September 2026).

## T20 · Li (2005), p. 5

朝起早，夜眠迟；老易至，惜此时

**Translation:** Rise early in the morning and sleep late at night; old age comes easily, so cherish this time.

**Value / form:** Diligence; routine with rationale. Authority / reciprocity: Learner self-conduct; authority not explicit. Sanction / affect: No penalty or prohibited feeling. Reasons / qualification: Passing time is an explicit reason.

**Conditional mapping / counter-reading:** A rigid timetable is a possible enactment. The wording does not establish a medically appropriate sleep schedule or effects on autonomy.

Scan checked: printed p. 5; PDF image 15 (21 September 2026).

## T21 · Li (2005), p. 6

衣贵洁，不贵华；上循分，下称家

**Translation:** Value cleanliness in clothing, not splendor; conform to one’s station and be suited to the family’s means.

**Value / form:** Cleanliness/moderation; advice and contrast. Authority / reciprocity: Station/family circumstances matter; reciprocity unstated. Sanction / affect: No explicit sanction or feeling rule. Reasons / qualification: Family means qualify dress.

**Conditional mapping / counter-reading:** Standardization requires moralizing one permitted form beyond relevant circumstances. Proportionate spending and cleanliness are counter-readings.

Scan checked: printed p. 6; PDF image 16 (21 September 2026).

## T22 · Li (2005), p. 6

对饮食，勿拣择；食适可，勿过则

**Translation:** With food and drink, do not be choosy; eat an appropriate amount and do not exceed the rule or measure.

**Value / form:** Moderation; prohibition. Authority / reciprocity: Learner conduct; authority not explicit. Sanction / affect: No explicit sanction or affect rule. Reasons / qualification: Appropriateness/limit provides qualification.

Conditional mapping / counter-reading: Rigid enforcement could constrain discretion only under additional enactment conditions. Appropriate portions and consideration of need remain constructive readings; the wording alone supplies no nutritional or autonomy outcome.

Scan checked: printed p. 6; PDF image 16. The edition prints 对饮食，勿拣择；食适可，勿过则. The graph 则 is retained and rendered as rule or measure; 勿过量 is not substituted. Other-edition variation is not assessed here.

## T23 · Li (2005), p. 6

步从容，立端正；揖深圆，拜恭敬

**Translation:** Walk unhurriedly and stand upright; bow deeply and fully, and make obeisance respectfully.

**Value / form:** Comportment; instructions. Authority / reciprocity: Conduct convention; authority not explicit in these lines. Sanction / affect: Respectful bearing; penalty unstated. Reasons / qualification: No stated exception or rationale.

**Conditional mapping / counter-reading:** Standardization requires insistence despite justified alternatives. The form alone may express courtesy and does not prove pressure or concealed affect.

Scan checked: printed p. 6; PDF image 16 (21 September 2026).

## T24 · Li (2005), p. 6

将入门，问孰存；将上堂，声必扬

**Translation:** Before entering a doorway, ask who is there; before entering a hall, announce one’s presence aloud.

**Value / form:** Consideration; entry protocol. Authority / reciprocity: Entrant attends to occupants; reciprocity unstated. Sanction / affect: No explicit sanction or feeling rule. Reasons / qualification: Entering a space specifies context.

**Conditional mapping / counter-reading:** Rigid form is possible, but privacy, coordination, and avoiding surprise are plausible purposes. Speaking audibly complicates a universal silence interpretation.

Scan checked: printed p. 6; PDF image 16 (21 September 2026).

## T25 · Li (2005), p. 7

话说多，不如少；惟其是，勿佞巧

**Translation:** It is better to speak little than too much; say only what is true or fitting, and do not use glib or artful words.

**Value / form:** Truthfulness/restraint; comparison and prohibitions. Authority / reciprocity: Speaker not explicitly age-defined. Sanction / affect: Excess/artifice discouraged; feeling not prohibited. Reasons / qualification: Truth/fittingness qualifies restraint.

**Conditional mapping / counter-reading:** Standardization requires a generalized quietness rule. Suppression needs separate affect evidence; necessary inquiry, disclosure, and correction are counter-cases.

Scan checked: printed p. 7; PDF image 17 (21 September 2026).

## T26 · Li (2005), p. 7

见未真，勿轻言；知未的，勿轻传

**Translation:** If you have not clearly seen, do not speak lightly; if you do not know precisely, do not pass it on lightly.

**Value / form:** Epistemic caution; prohibitions. Authority / reciprocity: Knower/speaker; adult authority not specified. Sanction / affect: No penalty or feeling rule. Reasons / qualification: Uncertain evidence qualifies speech.

**Conditional mapping / counter-reading:** Counter-resource to unexamined assertion. It should not be recoded as silencing simply because it limits unsupported claims.

Scan checked: printed p. 7; PDF image 17 (21 September 2026).

## T27 · Li (2005), p. 8

闻誉恐，闻过欣；直谅士，渐相亲

**Translation:** Be apprehensive on hearing praise and pleased on hearing criticism; upright and trustworthy people will gradually draw near.

**Value / form:** Receptivity to correction; affective counsel. Authority / reciprocity: Learner and candid others; age not explicit. Sanction / affect: Apprehension/pleasure prescribed; social consequence. Reasons / qualification: Good companions supply a stated consequence.

**Conditional mapping / counter-reading:** Affect moralization is possible if ambivalence becomes illegitimate. Openness to criticism also counters claims of moral infallibility.

Scan checked: printed p. 8; PDF image 18 (21 September 2026).

## T28 · Li (2005), p. 8

无心非，名为错；有心非，名为恶

**Translation:** Unintentional wrongdoing is called a mistake; intentional wrongdoing is called an evil.

**Value / form:** Intention-sensitive judgment; distinction. Authority / reciprocity: Agent/evaluator not age-specific. Sanction / affect: Different moral labels; no specific penalty or feeling rule. Reasons / qualification: Intention explicitly qualifies judgment.

**Conditional mapping / counter-reading:** Counter-case to indiscriminate moral blame. It does not establish a contemporary theory of culpability or child outcomes.

Scan checked: printed p. 8; PDF image 18 (21 September 2026).

## T29 · Li (2005), p. 8

过能改，归于无；倘掩饰，增一辜

**Translation:** If a fault can be corrected, it returns to nothing; if it is concealed, another wrong is added.

**Value / form:** Correction/honesty; conditional consequences. Authority / reciprocity: Wrongdoer not age-specific; reciprocity possible. Sanction / affect: Concealment compounds fault; no feeling required. Reasons / qualification: Correction and concealment differentiate cases.

**Conditional mapping / counter-reading:** Counter-resource supporting fallibility and repair. Pressure is possible only with further enacted contingencies, not entailed by correction itself.

Scan checked: printed p. 8; PDF image 18 (21 September 2026).

## T30 · Li (2005), p. 8

凡是人，皆须爱；天同覆，地同载

**Translation:** All people should be treated with care; the same heaven covers them and the same earth bears them.

**Value / form:** Universal care; injunction with shared-world rationale. Authority / reciprocity: All people included, not only elders. Sanction / affect: No explicit sanction or feeling rule. Reasons / qualification: Shared heaven/earth supplies a reason.

**Conditional mapping / counter-reading:** Counter-resource to selective concern. Any autonomy benefit remains hypothetical and depends on how care and participation are enacted.

Scan checked: printed p. 8; PDF image 18 (21 September 2026).

## T31 · Li (2005), p. 9

将加人，先问己；己不欲，即速已

**Translation:** Before doing something to another, first ask yourself; if you would not want it, stop at once.

**Value / form:** Reciprocity; reflective test. Authority / reciprocity: Potentially mutual rather than age-exclusive. Sanction / affect: Self-restraint, not a specified external penalty. Reasons / qualification: Perspective-taking and preference qualify action.

**Conditional mapping / counter-reading:** Counter-resource to unilateral authority. Applying it to both adults and children is a proposed reconstruction, not an observed lesson.

Scan checked: printed p. 9; PDF image 19 (21 September 2026).

## T32 · Li (2005), p. 9

势服人，心不然；理服人，方无言

**Translation:** If power makes people submit, their hearts do not assent; if reason convinces them, only then is there no objection.

**Value / form:** Reason over force; contrast. Authority / reciprocity: Power is not sufficient for inward assent. Sanction / affect: Outward submission distinguished from inward response. Reasons / qualification: Reason is contrasted with force.

**Conditional mapping / counter-reading:** Counter-resource to equating compliance with endorsement. Silence still cannot be used as a contemporary measure of agreement.

Scan checked: printed p. 9; PDF image 19 (21 September 2026).

## T33 · Li (2005), p. 10

不力行，但学文，长浮华，成何人！但力行，不学文，任己见，昧理真

**Translation:** Without putting learning into practice, merely studying texts increases superficial display—what sort of person does one become? Merely acting without studying texts leaves one to personal opinions and obscures the truth of principles.

**Value / form:** Learning/practice balance; paired criticism. Authority / reciprocity: Learner conduct; adult status not explicit. Sanction / affect: Superficiality or error named; no specific affect rule. Reasons / qualification: Both one-sided approaches are criticized.

**Conditional mapping / counter-reading:** Counter-resource to reducing instruction to recitation or blind action. It does not establish a tested educational method.

Scan checked: printed p. 10; PDF image 20 (21 September 2026).

## T34 · Li (2005), p. 10

心有疑，随札记；就人问，求确义

**Translation:** When doubts arise, note them down; consult someone and seek the exact meaning.

**Value / form:** Inquiry/accuracy; invitation/instruction. Authority / reciprocity: Knowledgeable others assist; infallibility not explicit. Sanction / affect: No explicit sanction or feeling rule. Reasons / qualification: Doubt prompts inquiry.

**Conditional mapping / counter-reading:** Counter-resource to interpretive monopoly. Inquiry may retrieve a fixed answer or revise understanding; enactment and child reception must distinguish them.

Scan checked: printed p. 10; PDF image 20 (21 September 2026).

# S4. Contextual and psychological evidence register

This register identifies the limited evidence used to motivate the revised contextual and mechanism claims. It is not a systematic review or an exhaustive listing of all relevant studies. Full bibliographic entries are in the manuscript’s References.

## Zhang (2019), printed pp. 146–148

**Access basis:** Recovered full chapter

**Permitted use and boundary:** Documentary overview of uses in schools, recitation initiatives, community/training settings, and families. Not a current census, representative observation, or source of voluntariness rates.

## Lin (2018)

**Access basis:** Retained project evidence record; bibliographic identity checked against institutional records

**Permitted use and boundary:** One third-grade, eleven-week, multicomponent action-study context; recitation, explanation, stories, role play, and responsibility activities. No isolated text effect, randomized comparison, or autonomy outcome is inferred. The manuscript omits a participant-count detail because the earlier record notes an internal count discrepancy.

## Yao and Wong (2021)

**Access basis:** Abstract only

**Permitted use and boundary:** Four fifth-grade classes, N = 242, with reported prosocial and relationship outcomes. Allocation, fidelity, detailed methods, and causal strength are not assessed. Those outcomes are not relabeled autonomy.

## Assor et al. (2004); Roth et al. (2009); Roth and Assor (2010)

**Access basis:** Previously audited psychological sources retained

**Permitted use and boundary:** Support considering conditional regard and regulation/emotion pathways in other populations and tasks. They neither test this primer nor establish transfer to the proposed approximately 8–12-year target.

## Zhu et al. (2023)

**Access basis:** Previously verified full-text study retained

**Permitted use and boundary:** Chinese psychological-control measurement with adolescent samples; cross-sectional convenience-sample limits retained. Not a validated middle-childhood or teacher-control instrument and not causal validation of this model.

# S5. Secondary direct-quotation locator register

These are the direct-quotation locators retained from the earlier source audit, plus the explicit removal of the Mercer direct quotation. They are not a claim that every historical source was newly re-audited in facsimile during this revision. Chinese-source translations are attributed to the authors; normative quotations do not establish educational effects.

## Huang (2011), p. 49

“grounded in children’s everyday lives”

Author’s translation; printed page corrected in the earlier audit, not PDF physical page 57.

## Qin (2018), p. 36

“should be approached dialectically”

Author’s translation; retained printed-page correction, not the earlier physical-page locator.

## Shen (2018), p. 10

“in a one-way, monologic manner”

Author’s translation; pedagogical critique, not evidence of prevalence or child effects.

## Zhang (2019), p. 164

“should not be compulsory”

Author’s translation; a recommendation, not a finding that participation is generally compulsory.

## Bai (2014), p. 37

“equal dialogue and emotional communication”

Author’s translation; normative proposal, not an intervention result.

## Cam (2014), p. 1209

“good judgement through collaborative inquiry”

Conceptual aim; the previous additional quotation at p. 1208 was removed when the framework was narrowed.

## Mercer et al. (2019)

Direct quotation removed; conceptual paraphrase retained

The earlier text cited manuscript pagination rather than journal pages. The revision uses paraphrase without converting those locators into guessed printed pages.

# S6. Newly added conceptual sources and access limits

## Chirkov et al. (2003)

Full text consulted; conceptual distinction between autonomy, individualism, and independence. Cross-cultural findings are not validation in Chinese children.

https://selfdeterminationtheory.org/SDT/documents/2003_ChirkovRyanKimKaplan.pdf

## Kağıtçıbaşı (2005)

Publisher abstract and bibliographic record consulted. Used only for its explicit conceptual distinction between agency and interpersonal distance; no detailed evidence or direct quotation is taken from the unavailable full text.

https://journals.sagepub.com/doi/10.1177/0022022105275959

Source bibliography: the manuscript’s References provides the complete bibliographic records. The Chirkov DOI is 10.1037/0022-3514.84.1.97; the Kağıtçıbaşı DOI is 10.1177/0022022105275959. Access levels are deliberately different and are not collapsed into a claim of full-text verification for both sources.
